# Multifaceted analysis of training and testing convolutional neural networks for protein secondary structure prediction

**DOI:** 10.1371/journal.pone.0232528

**Published:** 2020-05-06

**Authors:** Maxim Shapovalov, Roland L. Dunbrack, Slobodan Vucetic

**Affiliations:** 1 Fox Chase Cancer Center, Philadelphia, PA, United States of America; 2 Temple University, Philadelphia, PA, United States of America; Linköping University, SWEDEN

## Abstract

Protein secondary structure prediction remains a vital topic with broad applications. Due to lack of a widely accepted standard in secondary structure predictor evaluation, a fair comparison of predictors is challenging. A detailed examination of factors that contribute to higher accuracy is also lacking. In this paper, we present: (1) new test sets, *Test2018*, *Test2019*, and *Test2018-2019*, consisting of proteins from structures released in 2018 and 2019 with less than 25% identity to any protein published before 2018; (2) a 4-layer convolutional neural network, *SecNet*, with an input window of ±14 amino acids which was trained on proteins ≤25% identical to proteins in *Test2018* and the commonly used *CB513* test set; (3) an additional test set that shares no homologous domains with the training set proteins, according to the Evolutionary Classification of Proteins (ECOD) database; (4) a detailed ablation study where we reverse one algorithmic choice at a time in *SecNet* and evaluate the effect on the prediction accuracy; (5) new 4- and 5-label prediction alphabets that may be more practical for tertiary structure prediction methods. The 3-label accuracy (helix, sheet, coil) of the leading predictors on both *Test2018* and *CB513* is 81–82%, while *SecNet*’s accuracy is 84% for both sets. Accuracy on the non-homologous ECOD set is only 0.6 points (83.9%) lower than the results on the *Test2018-2019* set (84.5%). The ablation study of features, neural network architecture, and training hyper-parameters suggests the best accuracy results are achieved with good choices for each of them while the neural network architecture is not as critical as long as it is not too simple. Protocols for generating and using unbiased test, validation, and training sets are provided. Our data sets, including input features and assigned labels, and *SecNet* software including third-party dependencies and databases, are downloadable from dunbrack.fccc.edu/ss and github.com/sh-maxim/ss.

## Introduction

The prediction of secondary structure—alpha helices, beta sheet strands, and coil regions—is a long-standing problem in computational biology [1]. Given an amino-acid sequence, the task is to predict a sequence of the same length that designates the secondary structure defined by an alphabet of a certain size. The commonly used *DSSP* program [2] designates the secondary structure of experimental structures with an 8-letter alphabet (*H*, *B*, *E*, *G*, *I*, *T*, *S*), which can be reduced to a smaller alphabet (commonly *H*, *E*, *C*) according to defined rules. The results of secondary structure prediction have often been used as inputs to tertiary structure prediction methods [3–9], estimation of folding rates [10], prediction of solvent exposure of amino-acid residues [11–13], prediction of beta-turn locations and types [14–16], discrimination of intrinsically disordered regions [17, 18], accurate multiple sequence alignment [19–22], protein function prediction [23, 24], and prediction of missense mutation phenotypes [25].

The problem of predicting secondary structure from sequence has a long history, starting in 1965 [26]. Over a period of more than 50 years, the accuracy has gradually increased due to an increase in the number of experimentally determined structures in the Protein Data Bank (PDB), the amount of sequence information available for the determination of features derived from multiple sequence alignments, computational power, and the evolution of machine learning algorithms. Structures in the PDB provide information for deriving the ground-truth secondary structure labels used for training and testing of predictive models. It currently contains more than 150 thousand experimental structures and about 15 thousand non-redundant proteins (with <25% mutual sequence identity) [27]. The number of available protein sequences in sequence databases has dramatically increased from 5 million in 1999 when the widely-used *PSIPRED* was published [28] to 168 million today.

Secondary structure prediction methods and our ability to benchmark them have been evolving and improving too. Several reviews [1, 19, 29–33] provide an excellent source of the history of improvements in prediction methods. These methods have been divided into a set of four generations of methods covering the 50-year time period from 1960 to 2010 [1, 30, 34]; we also suggest a new fifth generation from the 2010s until today and describe it in more detail.

The *first generation* of methods—such as *C+F* [35], *Lim* [36], and *GORI* [37]—from the 1960s and 70s relied on single amino-acid preferences for the *H*, *E*, and *C* secondary structure types. In 1983 the original accuracy of 65–70% reported by the first-generation methods was revised downwards to 48–56% [38]. Dickerson et al. were the first to demonstrate that evolutionary information was helpful for secondary structure prediction [39]. However, the evolutionary approach during this period was limited by the small number of homologous protein sequences available for any target.

The *second-generation* methods from the 1980s and early 1990s—such as *Schneider* [40], *ALB* [41], *GORIII* [42], *COMBINE* [43], and *S83* [44]—utilized statistical analysis of a stretch of adjacent residues to predict secondary structure of a central residue [30, 34]. These were based on statistical information, sequence patterns, physiochemical properties, neural networks (NN), graph theory, multivariate statistics, expert rules, and nearest-neighbor algorithms [34]. The second-generation methods benefited from larger sequence databases and usage of evolutionary information. For instance, Zvelebil et al. incorporated evolutionary information in their method by predicting secondary structure for each protein in an alignment and then reporting an average prediction [45]. However, the second generation methods saturated at a low 3-label accuracy of 58–63% and experienced two problems: 1) beta strands were predicted at very low accuracy levels of 28–48%, marginally better than random; 2) predicted helices and strands were too short to be practical [34].

The methods of the *third generation* from the mid-1990s—such as *PHD* [46], *LPAG* [47], *SSP* [48]—achieved a 10 percentage-point improvement in the 3-label accuracy over second-generation methods, leading to 68–72% accuracy. Methods from the late 1990s to the early 2000s, including *PSIPRED* [49], *JPred2* [50], *SSpro* [51], and *PROF* [52], further reached 75–77%. The success came from three factors [30, 34]: 1) use of evolutionary information encoded in a multiple-sequence alignment (MSA) or position specific scoring matrix (PSSM) as direct inputs to a prediction program; 2) larger databases than for the second-generation methods; and 3) more advanced algorithms. The algorithms based on neural networks [30, 46, 49, 53, 54] demonstrated the greatest improvement.

Inspired with evolutionary information features as input in past applications, the *fourth-generation* methods from mid-late 2000s further utilized a variety of additional input features [33] including penta-peptide statistics [55, 56], conserved domain profiles [57], frequent amino-acid patterns [58], predicted torsion angles [59, 60], predicted residue contact maps [61], predicted residue solvent accessibility [62], predicted tertiary structure [62, 63], and predicted pseudo-energy parameters for helix formation [64]. Most of these individually led to small improvements. For example, Meiler et al. suggested seven representative amino-acid properties as input and showed their tenfold cross-validated accuracy increased only by 0.5 percentage points from 77% [65]. The predicted residue solvent accessibility improved the 3-label accuracy by 3 points [66]. The predicted dihedral angles helped by 2 points [67]. However, these features together did not improve the 3-label accuracy beyond 80% [68]. Some of the fourth-generation methods—such as *HYPROSP* [69], *PROTEUS* [70], *MUpred* [71], and *DISTILL* [72]—took advantage of structural fragments or templates of homologous sequences to attempt a breakthrough. It only brought a modest 3 percentage-point improvement and did not raise the 3-label accuracy beyond 80% with or without the additional input features.

We have identified 69 papers published in the last 10 years about secondary-structure prediction methods. Some of these methods we assign to a new *fifth-generation* if they have been developed with more sophisticated neural network architectures, such as deep convolutional neural networks (CNN), bidirectional recurrent NNs such as long short-term memory, and residual NNs and their combinations, [73–82] and/or incorporated more advanced evolutionary input features such as pairwise co-evolutionary models [74, 76–78, 82]. Some of them also revisited the template-based approach [73, 83] or employed an ensemble of models with an average vote [73, 74, 78, 80, 82, 84] or were trained on predicted residue contacts, residue surface accessibility, Cα-atom exposure, backbone torsion angles, and 3-label secondary structure predictions from other programs [82]. The template-free [74–81, 83, 84] methods reported accuracies from 82 to 87%, a substantial improvement over the fourth-generation accuracy of 80%. The theoretical 3-label accuracy limit is 90–95% [1, 30, 72, 82, 85, 86], based on the rate at which different programs such as *DSSP* and *Stride* [87] assign the same secondary structure to experimental coordinates.

In 2015, the fifth-generation method *SPIDER2* [81, 88] utilized three iteratively connected CNNs, each consisting of three hidden layers, achieving a 3-label accuracy of 82%. *Jpred4* [74] had 82% accuracy again in the same year by combining several CNNs trained on the same multiple sequence alignments presented to the networks in different ways. One year later in 2016, *DeepCNF* [75] achieved a 2 percentage-point accuracy improvement to 84% by combining a 5–7 layer CNN with conditional random field as an additional layer. The CNN was constructed in a funnel style by enforcing the same weights in neighboring input and hidden nodes and thus limiting the total number of parameters to train and allowing for longer-range sequence information. The final conditional random field layer was used to account for correlation of adjacent residues. In 2017, the authors of *SPIDER2* upgraded their previous method to a new NN architecture in *SPIDER3* [76], relying on four hidden layers with the first two bidirectional recurrent NN (BRNN) layers followed by two fully connected layers, and reporting an 84% 3-label accuracy, an improvement of 2 percentage points over *SPIDER2*. Also in 2017, *FSVM* [83] was reported with an 83% template-free accuracy achieved with a fuzzy support vector machine. In 2018, *MUFOLD-SS* [77] reported a 3-label accuracy of 84% with a neural network relying on nested inception models which are nested networks of several parallel CNNs that proved to be state-of-the-art in image recognition. At the same time, *PORTER5* [78] achieved 84% accuracy again by employing an ensemble of BRNNs. In 2018, another method, *PSRSM* [84] reported an improvement of 1–2 points to 85-86% accuracy; however, in an independent study the reported accuracy was revised downward to 82% [82]. The *PSRSM* publication does not mention a sequence similarity exclusion between training and test sets, which could be a cause for this overestimation. The 2018 method, *CNNH_PSS* (a 5-layer multiscale CNN with highways between neighboring layers), was only trained for 8 labels and reported 70% accuracy [79]. Another 2018 method, *eCRRNN*, an ensemble of 10 networks based on a combination of convolutional, residual, and bidirectional recurrent NNs), claimed the highest 8-label and 3-label accuracies to-date of 74% and 87% respectively [80].

The fifth-generation methods [73–84] all depend on multiple sequence alignments of the target sequences, while several of these methods also rely on templates [73, 83], i.e., real protein structures from a training set consisting of experimental coordinates of protein fragments. These “template-based” methods can achieve higher accuracies, in the range 86–93%, than template-free methods. However, homologous structures are not available for many proteins that would be targets of secondary structure prediction, and such methods are not solutions to the general secondary structure prediction problem. The accuracy of the template-based methods drops from 86–93% to 80–83% without templates and further down to 74–77% without homologous sequences [73, 82, 83]. Similarly, the accuracies of the template-free MSA-based methods drop from 82–84% to 73–75% without the use of homologous sequences [75].

The template-based *SSpro5* from 2014 [73] utilizes a *BLAST* [89] search of the PDB to find similar sequence fragments of at least length 10 to a target sequence and reports the most common *DSSP*-assigned class in the set of proteins selected for a given position. When no similar sequences or no dominant *DSSP* class in the similar sequences are found, secondary structure prediction is based on an ensemble of 100 BRNNs trained on the data set. With templates, *SSpro5* achieves 93% 3-label accuracy; without templates *SSpro5* has only 79–80% accuracy. *FSVM* [83] described above can also run in a template-based mode by using the same sequence-based structural similarity concept as in *SSpro5*. In this mode, *FSVM*’s 3-label accuracy increases from 83% to 93%.

One common issue that has recurred repeatedly during the history of secondary structure prediction is that reported accuracies have not always been upheld when methods were applied to new benchmark test sets [30, 34, 38, 46, 82, 90]. Rost and Sander [34] state that overoptimistic claims are caused by inadequate quality and size of test sets not meeting several requirements. First, significant pairwise sequence identity cannot be observed between proteins used for training and test set [90, 91]. For example, in [84] training was performed on a set representing the entire sequence space of the PDB leading to very similar proteins in training and testing sets. Second, the size of a test set must be greater than few tens of proteins [46]. Third, due to varying structural complexity with certain features easier to predict, all available unique proteins must be used for testing. Inadvertently probing a test set few or several times for development decisions may also be a source of accuracy overestimation [34]. It is challenging to compare programs on common benchmark sets because the programs are trained on different sets of structures, some of which may be in any particular benchmark. For example, the *CB513* data set [91] has been used repeatedly [75, 77, 79, 80, 83], even though related proteins may be used in the training data for a new program [73, 76, 78, 82].

In this paper, we investigate factors that contribute to the accuracy of template-free protein secondary structure with (1) an ensemble of 10 simple traditional 4-layer CNNs and (2) rigorously defined and independent training, validation, and test sets. We developed several test sets and describe how to benchmark existing or future methods against the new test sets (or similarly constructed test sets in the future) and what mistakes to avoid during training and testing.

Our main test set, *Test2018*, consists of proteins whose structures were determined in 2018 that do not share more than 25% sequence identity with any structure of any resolution or experiment type deposited before January 1, 2018. This enables us to compare our program, *SecNet*, with methods that were trained prior to the beginning of 2018. While there has been only ~5 percentage-point improvement from third-generation methods of the early 2000s, we demonstrate an accuracy increase of 2–3 percentage points in both the 3- and 8-label accuracies compared to two recently developed deep learning models which had reported the highest accuracy on the popular *CB513* data set available since 1999.

The 25–30% sequence identity cutoff between training and testing sets has been common practice for many years [42, 46, 48, 69, 75, 79, 92]. We developed three additional testing sets where we enforced stricter criteria that would eliminate most if not all evolutionary relationships between our training/validation set and the testing set. For example, we used ECOD (Evolutionary Classification of Domains) to develop a test set of proteins that do not share any homologous domain with a protein in our training set. ECOD clusters domains at the homology level (“H level”) even if the domains are very remotely related and have different topologies (usually sharing a common core and some functional similarity as evidence of a common ancestor). Our secondary structure prediction accuracy only drops by one percentage point when these stricter criteria are enforced.

Through an ablation study that followed *SecNet* development, we investigated factors that are important for high-accuracy prediction such as method complexity, types of input features, window size, database source and size, alignment parameters, and training hyper-parameters. For example, contrary to many methods that use extremely wide sequence windows (as long as the whole sequence) [75, 76, 80, 93], our results show that CNNs do not benefit in terms of overall accuracy beyond 15 residues away from a prediction label. We discuss the prediction practicality of secondary structure labels for protein tertiary structure prediction, and propose new 4 and 5 prediction label schemes that should be more useful for structural biology.

## Results

### Data sets for training, validation, and testing secondary structure prediction methods

**Fig 1:**
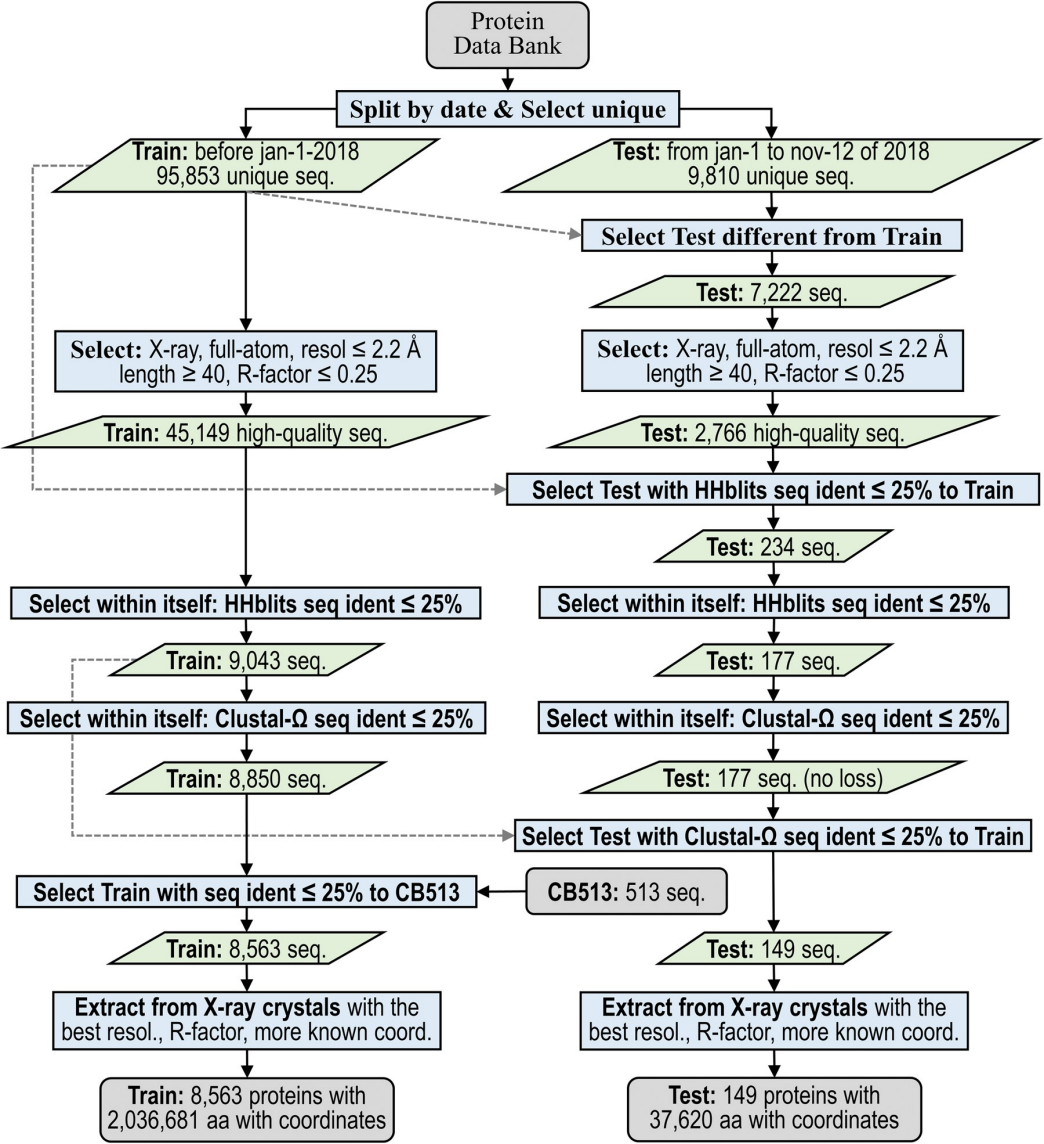
Protocol flowchart for preparation of 2018 unbiased test and training sets (*Set2018* dataset). Protocol input and output are shown with gray rounded rectangles. All action processes are in blue rectangles. Green parallelograms represent intermediate input and output data. The split date for *Set2018* training and test sets is Jan 1, 2018. By using two sequence alignment programs, the protocol removes from the test set any proteins with more than 25% sequence identity to any previously published PDB structure of any experimental type, resolution, or quality. The test set guarantees unbiased accuracy estimation for our prediction method, *SecNet*, and any previous software trained and validated on proteins released before Jan 1, 2018.

We produced separate sets of structures from the PDB for our training, validation, and test data sets with the protocol shown in Fig 1 and fully described in *Methods*. Our aim was to make the protocol reproducible, so that it can be reused for creation of new test sets in the future. Because we wanted to compare our secondary structure prediction program with the results in earlier publications, we developed a test set that would not have proteins in the training and testing data of earlier programs. Similar approaches for deriving test sets have been used by others [82].

To develop the test set, we placed all 9,810 unique protein chains in PDB entries of any resolution or experimental type released between January 1 and November 12, 2018 into a starting set. We filtered the test proteins to satisfy a 2.2 Å resolution threshold, a 0.25 R-factor cutoff consistent with this resolution [94], and a minimal chain length of 40 residues. From this reduced set, we additionally removed any sequence with more than 25% sequence identity to any of the 95,853 unique sequences in structures released before January 1, 2018 of any resolution, any experiment type, and any quality-control characteristics. This similarity exclusion took place if either of two programs, *HHblits* [95] or *Clustal-Omega* [96], calculated a pairwise sequence identity above the 25% cutoff (the exact definition of the sequence identities by *HHblits* and *Clustal-Omega* in *Methods*). Finally, we applied the same pairwise sequence identity cutoff of 25% according to the same two alignment programs within the test set itself. In the last step, when several similar proteins had to be removed, the algorithm kept proteins with a higher resolution or better R-factor or more residues with known coordinates in that order [27].

For the training/validation set, the same resolution, R-factor, length, and sequence identity criteria were applied to the sequences and structures that were released by the PDB prior to 2018. Chains with sequence identity ≥ 25% with any protein in the popular *CB513* data set were also removed from the set so that *CB513* could also be used in testing (see below). The resulting set was split with a 9:1 ratio to produce the training and validation sets. We applied *DSSP* to the chains in each data set, producing training, validation, and test sets respectively that have 7707, 856, and 149 protein chains and 1830, 207, and 38 thousand amino acids of labeled secondary structure (Table 1 and Table A in S1 File). We refer to the three data sets collectively as *Set2018*, and the testing set individually as *Test2018*. We provide *Set2018* (S1 Dataset) including the PDB entry and chain, the full-length original and unmodified sequences using a standard 21-letter notation (with modified amino acids represented with single letters of their unmodified counterparts where possible; other non-standard amino acids are represented by “X”), and the *DSSP* codes (including the symbol “X” for residues with missing backbone coordinates).

**Table 1:** Statistics for the *Set2018* training, validation, and testing data sets.

Set2018			Proteins	Perc. of amino acids	No of amino acids
All = Train + Valid + Test			8,712	-	-
whole sequence			-	100.0	2,217,707
with coordinates			-	93.5	2,074,301
w/o coordinates			-	6.5	143,406
**All with coordinates**			**8,712**	**100.0**	**2,074,301**
H	α-helix		-	34.1	707,050
E	strand		-	22.3	463,310
C	coil		-	19.4	401,922
T	turn		-	11.1	229,566
S	bend		-	8.2	169,326
G	3_10_ helix or helix-3		-	3.9	80,536
B	β-bridge		-	1.1	22,245
I	π-helix or helix-5		-	0.017	346
**Training set**			**90% split of (All–Test) by proteins**		
Any			7,707	100.0	1,830,092
C	coil	Rule #1	-	38.6	706,364
H	helix		-	38.0	695,750
E	sheet		-	23.4	427,978
**Validation set**			**10% split of (All–Test) by proteins**		
Any			856	100.0	206,589
C	coil	Rule #1	-	38.7	79,985
H	helix		-	37.9	78,261
E	sheet		-	23.4	48,343
**Test set**			**Fixed, 2% of All**		
Any			149	100.0	37,620
C	coil	Rule #1	-	38.5	14,465
H	helix		-	37.0	13,921
E	sheet		-	24.5	9,234

Protein chain, amino-acid, 8-label, and *Rule #1* 3-label statistics for *Set2018* dataset with resolution of up to 2.2 Å. Training and validation sets were randomly divided with a 9:1 ratio from (All-Test) as full sequences for 10 cross-validation splits. The validation set statistics are shown for the second random seed split; it was used for design decisions, hyper-parameter tuning, and selection of the best model.

### SecNet: A 4-layer convolutional neural network for protein secondary structure prediction

**Fig 2:**
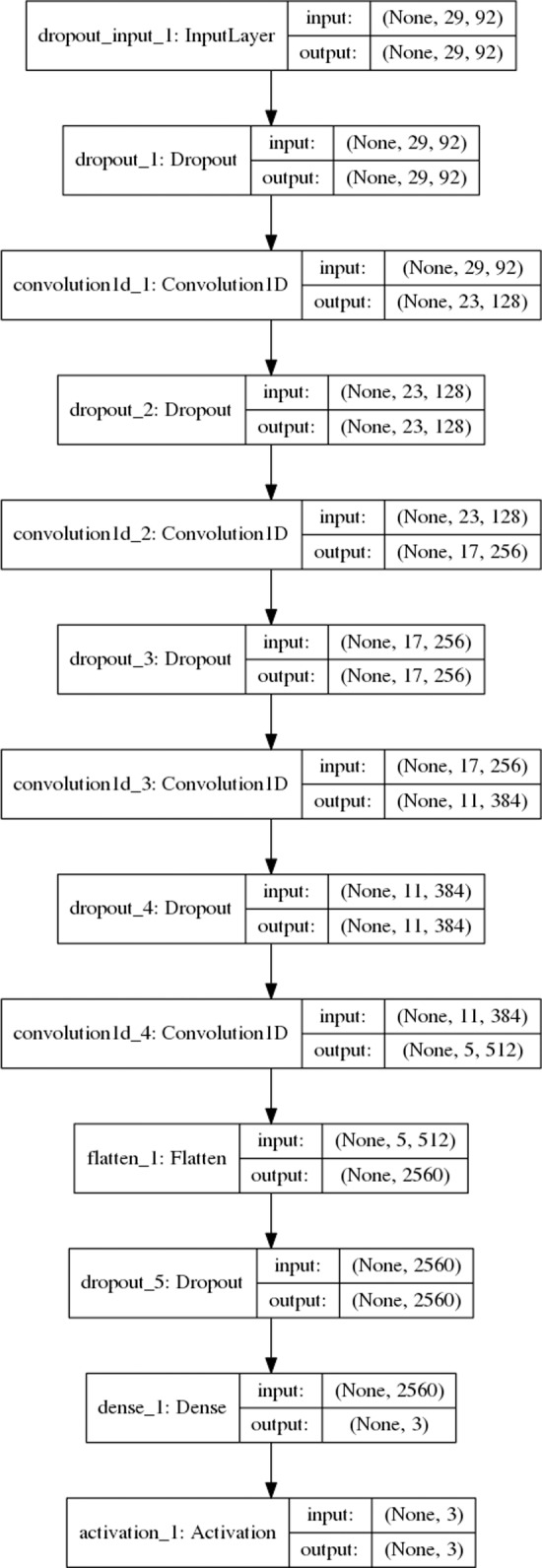
Architecture of *SecNet*. *SecNet* is a traditional CNN with an input layer, 4 hidden convolutional layers, and a dense layer with dropout regularization layers in between. The input layer reads a 29 x 92 matrix representing a sequence window of 29 amino acids centered on the one subject to prediction and 92 input features for all 29 positions. 92 features = one-hot encoding of 22 amino-acid types + 2 rounds x 20 *PSI-BLAST* profile values + 30 HMM alignment parameters. Each box encloses a linear dimensionality and number of features (2^nd^ and 3^rd^ values in parentheses) in input and output of each layer. The total number of parameters to train is about 2 million. The activation layer returns 3 probabilities for the 3 labels: *H*, *E*, and *C* of the central amino acid which are calculated with a *softmax* activation function. The label with the highest estimated probability is the predicted label.

We describe a traditional CNN with only 4 hidden layers (Fig 2), trained on our pre-2018 training set from *Set2018*. The validation set was used multiple times to try different architectures of convolutional and residual neural networks, to select features having a positive impact on prediction, and to optimize other hyper-parameters and NN trainable parameters themselves. (A detailed description of the optimization strategy of hyper-parameters is in *Methods*.) After the best model based on the highest validation accuracy during training was saved, the program was only once benchmarked on our unbiased *Test2018* test set. The 92 input features of our template-free method consist of one-hot encoding of an amino-acid sequence, two *PSI-BLAST* profiles (after 1 and 2 rounds of search on Uniprot90), and HMM parameters derived from a multiple sequence alignment of a target sequence and hits from the Uniprot20 sequence database [95]. We underline that our predictive model is a simple, traditional CNN, which is in contrast to the recent trend of using more and more complex methods and NN architectures [73–81]. It employs an input window of only 29 amino acids. Each sample has a depth of 92 features per amino acid; therefore, the dimensionality of the input to our NN is (29, 92) or 2,668 values in total to predict a single secondary-structure label of the central residue. The number of the labeled samples in the 2018 training set is 1,830,092 amino acids. A detailed description of our CNN and input features is provided in *Methods*.

We first tested the accuracy of predicting all 8 labels from *DSSP* (*H*, *E*, *C*, *G*, *I*, *S*, *T*, *B*); the 8-label accuracy is 73.0% on *Test2018*. To develop a program that is trained and tested on three labels (*H*, *E*, *C*), we need a rule to convert the 8-letter alphabet to the 3 letters. The most common rule, which we refer to as *Rule #1*, is (*H*, *G*, *I*) → *H*, (*E*, *B*) → *E*, (*T*,*S*,*C*) → *C* [97]. A less common rule, which we refer to as *Rule #2*, has a simpler definition: (*H*) → *H*, (*E*) → *E*, (*G*,*I*,*B*,*T*,*S*,*C*) → *C* has been used in some programs [53, 74, 80, 98–101]. Several published methods did not clearly define which 3-label rule was used [77, 78, 81, 83]. If we use *Rule #1* on the training, validation, and testing data, we achieve a 3-label accuracy of 84.0% on *Test2018*. The confusion matrices, true positive rates (recalls), positive predictive values (precisions), false negative rates, and false discovery rates for the 8-label and 3-label Rule #1 predictions are presented in Tables 2 and 3 respectively. When *SecNet* is trained, validated, and tested on data derived using *Rule #2*, which is easier to predict [28, 91], it has a higher accuracy of 86.0%.

**Table 2:** Confusion matrices for 8-label alphabet: *H*, *E*, *C*, *T*, *S*, *G*, *B*, and *I*. (1) Confusion matrix, (2) true positive rates (recalls) and false negative rates and (3) positive predictive values (precisions) and false discovery rates of *SecNet* on *Test2018* test set.

Confusion matrix of 37,620 test labels										
**Accuracy**		**Pred. freq.**	**True label**							
**73.0%**			**H**	**E**	**C**	**T**	**S**	**G**	**B**	**I**
**True freq.**		100%	33.26	23.31	19.27	10.94	8.24	3.70	1.24	0.05
**Predicted label**	**H**	36.88	**31.33**	0.40	1.34	2.15	0.51	1.03	0.09	0.03
	**E**	25.00	0.28	**19.95**	2.85	0.52	0.83	0.26	0.31	0.00
	**C**	23.18	0.86	2.38	**13.07**	1.88	3.59	0.81	0.60	0.00
	**T**	9.52	0.50	0.31	1.07	**5.71**	1.26	0.54	0.13	0.00
	**S**	3.40	0.04	0.22	0.74	0.37	**1.92**	0.07	0.04	0.00
	**G**	1.94	0.24	0.04	0.20	0.32	0.13	**0.99**	0.02	0.00
	**B**	0.06	0.00	0.01	0.01	0.00	0.00	0.00	**0.03**	0.00
	**I**	0.01	0.00	0.00	0.00	0.00	0.00	0.00	0.00	**0.01**
Column normalized table			**True label**							
			**H**	**E**	**C**	**T**	**S**	**G**	**B**	**I**
			100	100	100	100	100	100	100	100
**Predicted label**		**H**	**94.21**	1.72	6.95	19.61	6.20	27.87	7.53	66.67
		**E**	0.86	**85.57**	14.77	4.76	10.04	6.97	25.38	0.00
		**C**	2.59	10.22	**67.80**	17.15	43.58	21.77	48.60	0.00
		**T**	1.50	1.35	5.53	**52.16**	15.27	14.73	10.32	5.56
		**S**	0.11	0.95	3.85	3.38	**23.30**	1.94	3.44	0.00
		**G**	0.73	0.16	1.06	2.92	1.58	**26.65**	1.94	0.00
		**B**	0.01	0.03	0.04	0.02	0.03	0.07	**2.80**	0.00
		**I**	0.00	0.00	0.00	0.00	0.00	0.00	0.00	**27.78**
**Diagonal has True positive rates (Recalls)** Elsewhere False Negative Rates										
Row normalized table			**True label**							
			**H**	**E**	**C**	**T**	**S**	**G**	**B**	**I**
**Predicted label**	**H**	100	**84.94**	1.09	3.63	5.82	1.38	2.80	0.25	0.09
	**E**	100	1.14	**79.80**	11.39	2.08	3.31	1.03	1.25	0.00
	**C**	100	3.72	10.27	**56.37**	8.10	15.48	3.47	2.59	0.00
	**T**	100	5.25	3.30	11.20	**59.95**	13.21	5.72	1.34	0.03
	**S**	100	1.09	6.49	21.80	10.86	**56.40**	2.11	1.25	0.00
	**G**	100	12.44	1.92	10.53	16.41	6.70	**50.75**	1.23	0.00
	**B**	100	4.36	13.00	12.96	4.31	4.32	4.37	**56.68**	0.00
	**I**	100	0.00	0.00	0.00	0.00	0.00	0.00	0.00	**100.0**
**Diagonal has positive predictive values (Precisions)** Elsewhere False Discovery Rates										

**Table 3:** *Rule #1* of the popular 3-label (*H*, *C*, and *E*) alphabet: (1) confusion matrix, (2) true positive rates (recalls) and false negative rates and (3) positive predictive values (precisions) and false discovery rates of *SecNet*.

Confusion matrix of 37,620 test labels					
**Accuracy**^1^		**Pred. freq.**	**True label**		
**84.0%**			**C**	**H**	**E**
**True freq.**		100%	38.5	37.0	24.5
**Predicted label**	**C**	41.0	**32.1**	3.9	5.1
	**H**	36.8	3.5	**32.8**	0.4
	**E**	22.2	2.8	0.3	**19.1**
Column normalized table			**True label**		
			**C**	**H**	**E**
			100	100	100
**Predicted label**		**C**	**83.4**	10.4	20.6
		**H**	9.2	**88.7**	1.8
		**E**	7.4	0.9	**77.6**
**Diagonal has TPRs** Elsewhere FNRs					
Row normalized table			**True label**		
			**C**	**H**	**E**
**Predicted label**	**C**	100	**78.3**	9.4	12.3
	**H**	100	9.6	**89.2**	1.2
	**E**	100	12.8	1.5	**85.8**
**Diagonal has PPVs** Elsewhere FDRs					

The most common and harder 3-label (*H*, *C*, *E*) *Rule #1* is defined as (*G*, *I*) → *H*, (*B*) → E and (*B*, *S*) → C. Depending on different studies, it is 2–3 percentage points lower in accuracy than easier *Rule #2*.

^1^The overall accuracy is summation of the main diagonal of the confusion matrix.

We also tested *SecNet* with the popular *CB513* test set derived by Cuff and Barton in 1999 for benchmarking of competing secondary structure prediction software [91] and adopted for accuracy assessment many times [75, 77, 79, 80, 83]. *CB513* has several deficiencies. First, disordered residues that have no atoms present in the coordinates are deleted from the sequences and the DSSP strings altogether. This means that sequence profiles and HMMs will be distorted since proteins in the sequence database will seem to have insertions relative to the query protein even when they do not. Second, it has relatively poor average and maximal resolutions (2.11 Å and 3.5 Å) compared to sets that can be derived from the PDB today (e.g., the *Test2018* test set has average and maximal resolutions of 1.83 Å and 2.2 Å). The *CB513* data set protocol initially required ≤ 2.5 Å resolution; however, 5% of its entries have resolution between 2.5 and 3.5 Å resolution. Moreover, 32% of *CB513* entries have free R-factor in the worst 25^th^ percentile of the deposited structures in the PDB. Such models raise doubts about the quality of the refined structures [94]. Third, *CB513* has sequences of PDB chains that are fragmented into domains, which results in sequences 2.2 times shorter on average than their full PDB chains. *CB513* has sequences as short as 20 amino-acid residues; 5% and 20% of chains in *CB513* are shorter than 40 and 80 residues respectively. Finally, secondary structure labels were generated with an obsolete version of *DSSP* that was available in 1998.

Nevertheless, because we eliminated proteins from our training and validation sets with more than 25% sequence identity with any chain in *CB513* (Fig 1), we can fairly compare *SecNet* with numerous previous reports on *CB513*. We benchmarked *SecNet* on *CB513* and calculated 8-label, *Rule #1* 3-label, and *Rule #2* 3-label accuracies of 72.3%, 84.3%, and 86.3% respectively (Table 4), which are close to the *Test2018* accuracies with -0.7%, +0.3%, and +0.3% differences; a lower value for 8 *DSSP* labels may be related to the *CB513* deficiencies described above.

**Table 4:** Prediction accuracy of our *SecNet* software in 3 categories of labels on 5 test sets.

Training set	Testing set	SecNet Accuracy in 3 categories			Testing set size	
		8 labels	3 labels		#Proteins	#Amino acids
			harder Rule #1	easier Rule #2		
Set2018, 2.2Å	Test2018, 2.2 Å	73.0	84.0	86.0	149	37,620
	Test2019, 2.2 Å	74.0	84.7	86.5	220	50,590
	Test2018-19, 2.2 Å	73.7	84.5	86.2	352	80,666
	CB513	72.3	84.3	86.3	513	84,091
HigherRes, 1.8Å	HighRes, 1.8 Å	73.0	83.5	85.6	67	14,791
	CB513	72.0	84.0	86.0	513	84,091

Prediction accuracies are measured in 3 categories: 8 *DSSP* labels; the most common and harder 3-label *Rule #1*: (*H*, *G*, *I*) → *H*, (*E*, *B*) → *E*, (rest 4 labels) → C; and easier 3-label *Rule #2*: (*H*) → *H*, (*E*) → *E*, (rest 6 labels) → C. The 2.2 Å and 1.8 Å training sets have about 2 and 1 million residues respectively. The training and matching test sets share < 25% sequence identity in all cases. Six *SecNet* models were independently trained for 3 categories of labels x 2 training sets.

Concerned with discrepancies in X-ray crystal structures with resolution up to 2.2 Å in *Set2018* and up to 3.5 Å in *CB513*, we explored more stringent structural quality parameters that result in smaller training and test sets, but may result in higher prediction accuracy since secondary structures may be more accurately determined especially for 8 labels. To test this, we followed the same protocol to produce training, validation, and test sets with a 1.8 Å cutoff (*‘HigherRes*’ sets), and then retrained the *SecNet* neural network. We then benchmarked *SecNet* on the 1.8 Å *HigherRes* and *CB513* test sets. The 3-label accuracies are lower by 0.3–0.5 points for both test sets; 8-label accuracy is the same for the new test set compared to the 2.2 Å test set; it is lower by 0.3 points for *CB513* (rows 1, 4 vs. 5–6 in Table 4). The modest decreases are likely due to (1) the smaller data set size– 1,224,479 vs. 2,074,301 amino acids in the 1.8 Å and 2.2 Å data sets respectively and (2) a small impact from the ground-truth secondary-structure assignment at the higher resolution of 1.8 Å. Since *SecNet* has approximately the same accuracy on the 2.2 Å *Test2018* as it has on the 1.8 Å *HigherRes*, and since the 2.2 Å training and testing sets are much larger, all further results, discussion, and final benchmarking are reported on *Test2018* at 2.2 Å resolution.

We repeated the testing set preparation protocol for 2018–19 PDB entries with unchanged *Set2018* training/validation sets and generated *Test2019* and *Test2018-19* covering extra 14 months and total 24 months instead of 10 months of *Test2018*. For exact dates, please refer to *Methods*. Prediction accuracies for Test2019 and Test2018-19 are higher by 0.2–1.0 percentage points than for *Test2018* (Table 4) and suggest a higher proportion of harder prediction targets among the selected 2018 proteins than the selected 2019 proteins.

Next, we compared *SecNet* with 12 fifth-generation template-free methods with reported *CB513* accuracies in recent publications (Table 5). Our *SecNet* accuracies are 2–9 percentage points higher than 11 out of 12 previous methods with only *eCRRNN* reporting a higher accuracy by 1.7 points on 8-label predictions and 1.1 points higher on 3-label predictions. We chose to test *eCRRNN* directly as well as *DeepCNF*, both having the highest reported accuracies on *CB513*. *DeepCNF* uses a CNN with 5 layers and conditional random field as an additional layer and as input includes 42 features for each residue: 21 from a PSSM profile and 21 from one-hot encoding. *DeepCNF* has an effective window size of 51 amino acids. The *eCRRNN* architecture combines 6 blocks as a mixture of convolutional, residual and bidirectional recurrent NNs. The input to *eCRRNN* consists of 50 features for each residue– 20 values from a PSSM profile, 7 physical properties, a conservation score, and a set of twenty-two 22-dimensional orthogonal vectors. The second-block BRNNs process the full amino-acid sequence by reading all residues on the left and right from the residue subject to prediction, making the window width effectively unlimited.

**Table 5:** *CB513* accuracy of the 5^th^-generation template-free secondary-structure prediction methods.

Method	Seq id of CB513 and training data	8 labels			Harder 3-label Rule #1			Easier 3-label Rule #2		
		accuracy	source	diff	accuracy	source	diff	accuracy	source	diff
Our SecNet (2019)	< 25%	72.3	authors	0.0	84.3	authors	0.0	86.3	authors	0.0
**eCRRNN (2018)**	unspec	**74.0**→70.2	auth→calc	-2.1	81.2	calculated	-3.1	**87.3**→84.3	auth →calc	-2.0
MUFOLD-SS (2018)	unspec	70.6	authors	-1.7	-	-	-	82.7	eCRRNN	-3.6
CNNH_PSS (2018)	< 25%	70.3	authors	-2.0	-	-	-	-	-	-
DCRNN (2016)	< 25%	69.7	authors	-2.3	-	-	-	84.0	authors	-2.3
DCNN (2017)	< 25%	70.0	authors	-2.6	-	-	-	-	-	-
**DeepCNF (2016)**	< 25%	68.3→69.1	auth→calc	-3.2	**82.3**→81.8	auth→calc	-2.5	-	-	-
BLSTM (2015)	< 25%	67.4	authors	-4.9	-	-	-	-	-	-
GSN (2014)	< 30%	66.4	authors	-5.9	-	-	-	-	-	-
SSpro, free (2014)	Any	63.5	DeepCNF	-8.8	78.5	DeepCNF	-5.8	-	-	-
JPRED4 (2015)	Any	-	-	-	81.7	DeepCNF	-2.6	79.6	FSVM	-6.7
FSVM, free (2018)	< 25%	-	-	-	-	-	-	82.9	authors	-3.4
SPIDER2 (2015)	Any	-	-	-	-	-	-	81.2	FSVM	-5.1
PSIPRED (1999)	Any	-	-	-	79.2	DeepCNF	-5.1	-	-	-

*CB513* accuracies were compiled from recent papers of the fifth-generation, template-free methods. Accuracy taken from an original publication has “authors” tag. If a study did not utilize the *CB513* test set but a third-party benchmarked the method, the latter source is reported instead and the sequence identity to *CB513* is listed as “Any.” Some “authors” papers did not specify an identity cutoff between their training set and test sets, including *CB513* (“unspec”). The widely used *PSIPRED* program belongs to the third generation. We recalculated the *eCRRNN* and *DeepCNF* accuracies (“calc.”) with the original *CB513* (indicated after the →). *eCRRNN*, *DCRNN* and *BLSTM* employ bidirectional recurrent NNs; *MUFOLD-SS* applies an inception and residual NN; *CNNH_PSS* has a residual NN only; *GSN* uses a convolutional generative stochastic network; *DCNN* is a conditional residual CNN; *FSVM* stands for fuzzy SVM; the remaining methods are CNNs.

Since *DeepCNF* and *eCRRNN* were submitted for publication prior to the end of 2018, our *Test2018* set is independent of their training sets (at 25% sequence identity). We applied both methods to the *Test2018* test sequences, and determined that our *SecNet* has higher accuracies than *DeepCNF* by 3.3–3.4 percentage points (Table 6). Our method’s accuracies are 2.0–2.2% higher than *eCRRNN* on *Test2018*. Next, we ran both of these methods on the original *CB513* test set. The *DeepCNF* accuracies that we calculated on *CB513* closely reproduce those of the authors with slightly higher 8-label and slightly lower 3-label accuracies (Table 5). When the authors tested *DeepCNF* on *CB513*, they excluded 31 shorter entries, and this may account for these small observed differences. In contrast, our recalculated *CB513* accuracies for *eCRRNN* indicated that the authors overestimated their accuracy by 3.0–3.8 percentage points (Table 5). We excluded the possibility that we incorrectly executed the *eCRRNN* software by reproducing their results for the three tests sets included with their software. With these findings we updated the *CB513* accuracy for *eCRRNN* in Table 5 with our calculated values. With these accuracy revisions, *SecNet* is more accurate than *eCRRNN* by 2.0–2.2 points on *Test2018* and 2.0–3.1 points on *CB513*, depending on the label set.

**Table 6:** Benchmarking of *SecNet*, *DeepCNF*, and *eCRRNN* on *Test2018*.

Test set	Method	8 labels		3 labels			
				harder Rule #1		easier Rule #2	
		accur	diff	accur	diff	accur	diff
Set2018	**SecNet**	**73.0**	**0.0**	**84.0**	**0.0**	**86.0**	**0.0**
	eCRRNN	70.8	-2.2	81.8	-2.2	84.0	-2.0
	DeepCNF	69.6	-3.4	80.7	-3.3	-	-

The difference in accuracy of the earlier programs (*DeepCNF* and *eCRRNN*) may be due either to the NN architecture and training procedures, and/or the training and testing data, including the sequence database used to derive *PSI-BLAST* or HMM profiles. Because we have these data available from the authors of *DeepCNF*, we decided to investigate how well the *SecNet* architecture and training pipeline would behave with the training/testing data and sequence database used by the *DeepCNF* authors. In Table 7 we report these values when *DeepCNF* model with its original data set and sequence database chosen as a baseline. We re-trained the SecNet model for several combinations of the components and re-tested it on *Test2018* and *CB513*. It turns out that the major accuracy improvement comes from the optimized NN architecture and other hyper-parameters with 2.2–2.4 point and 1.9–2.2 point increases for *Test2018* and *CB513* respectively. Our sequence database is more recent (2017) than theirs (2015). A sequence database upgrade by 2 years from 2015 to 2017 brings additional 0.2–0.4 and 0.2 point improvements for *Test2018* and *CB513*. The expansion of the training data set from 5.6 to 8.6 thousand proteins of higher quality has a stronger rewarding effect of 0.4-0.5 and 0.4–0.9 points for *Test2018* and *CB513* respectively. Both our larger sequence database and large training data set bring a cumulative effect of 0.8–1.2 points and 0.6–1.0 points for *Test2018* and *CB513* which is equal or greater than a sum of individual effects. The accuracy increments in both testing sets and among 3 label types are consistent. The *Rule #2* 3-label results cannot be compared to *DeepCNF* baseline since it was not individually trained for that alphabet.

**Table 7:** Sources of *SecNet* accuracy improvement as measured on *Test2018* and *CB513*.

Training			Test2018					CB513				
Method	Dataset for training	Sequence database for train & testing	8 labels		3 labels			8 labels		3 labels		
					Rule #1		Rule #2			Rule #1		Rule #2
			accur	diff	accur	diff	accur	accur	diff	accur	diff	accur
DeepCNF	theirs	Theirs	69.6	0.0	80.7	0.0	N/A	69.1	0.0	81.8	0.0	N/A
**SecNet**	**theirs**	**Theirs**	**71.8**	**+2.2**	**83.1**	**+2.4**	**85.0**	**71.3**	**+2.2**	**83.7**	**+1.9**	**85.6**
SecNet	theirs	Ours	72.0	+2.4	83.5	+2.8	85.5	71.5	+2.4	83.9	+2.1	85.7
SecNet	ours	Theirs	72.3	+2.7	83.5	+2.8	85.4	72.2	+3.1	84.1	+2.3	86.2
SecNet	ours	Ours	73.0	+3.4	84.0	+3.2	86.0	72.3	+3.2	84.3	+2.5	86.3

The sources of *SecNet* accuracy improvement include (1) more suitable NN architecture, other hyper-parameters, and training pipeline, (2) a larger sequence database, and (3) a larger data set with stricter quality controls. These are measured by re-training *SecNet* using the data set and original sequence database from the *DeepCNF* original publication.

Several programs (including *eCRRNN*) in Table 5 do not specify a similarity cutoff (“unspec”) between training and test sets. Some apply a higher value [81, 92] than the commonly used 25%, and one program, *PSRSM* [84], did not mention any similarity exclusion technique between these sets in their publication. When training set proteins similar to the testing set are not excluded properly, it is likely that such methods overestimate their accuracy by overtraining. Accuracy may also be overestimated when a single data set is used as both validation set and testing set. To demonstrate this, we compared the accuracy on our testing set and our validation set achieved during training. As expected, the accuracy on the validation set is higher than the accuracy on the test set by 2.5% (75.5% vs. 73.0%) for 8-labels and 2.1% (86.1% vs. 84.0%) for *Rule #1* (Table 8). The accuracy on the training set is 6.8 points higher (79.8% vs. 73.0%) for 8 labels and 4.3 points higher (88.3% vs. 84.0%) for *Rule #1* 3-label predictions. The striking over-estimation for the validation and training sets demonstrates the importance of separate training, validation, and testing data sets. As another test, if we randomly permute proteins and split them into different validation and training sets with the 1:9 ratio many times (testing set is unchanged), individually train each, and select the best model based on the highest validation accuracy, we gain additional 2.3 and 2.0 point improvements in the 8-label and *Rule #1* 3-label validation accuracy with no improvement in the test accuracy (Table 8). In this case, the validation accuracy is boosted due to a favorable random re-distribution of proteins in the training and validation sets.

**Table 8:** Evidence of prediction accuracy overestimation of *SecNet* on *Set2018* validation and training sets relative to the test set.

	Set2018	Accuracy	
		SecNet	Diff. from Test
3 labels, Rule #1	Test	84.0	0.0
	Validation	86.1	+2.1
	Favorable Validation	88.0	+4.1
	Training	88.3	+4.3
8 labels	Test	73.0	0.0
	Validation	75.5	+2.5
	Favorable Validation	77.8	+4.8
	Training	79.8	+6.8

The test accuracy was probed only once for reporting accuracy results. Accuracy probes on the validation set were done multiple times for design decisions (~10s of times), hyper-parameter tuning (~100s), saving the best model during training (~100 probes per training x ~1,000s trainings = ~10,000s), and rejecting more complex *SecRes* residual NN (~10s). The “favorable validation” accuracy was achieved by splitting the whole training set with a 1:9 ratio into validation and reduced training sets more than 10 times (~100s). The models were individually trained, and the validation set with the best accuracy was chosen. The training set accuracy was probed few million times per training (~1,000,000s) during gradient-descent optimization. It is considerably below 100% owing to the regularization technique with 30% dropout. The overestimated numbers underline the importance of having separate test, validation, and training sets and probing the test set only once for reporting unbiased results.

**Fig 3:**
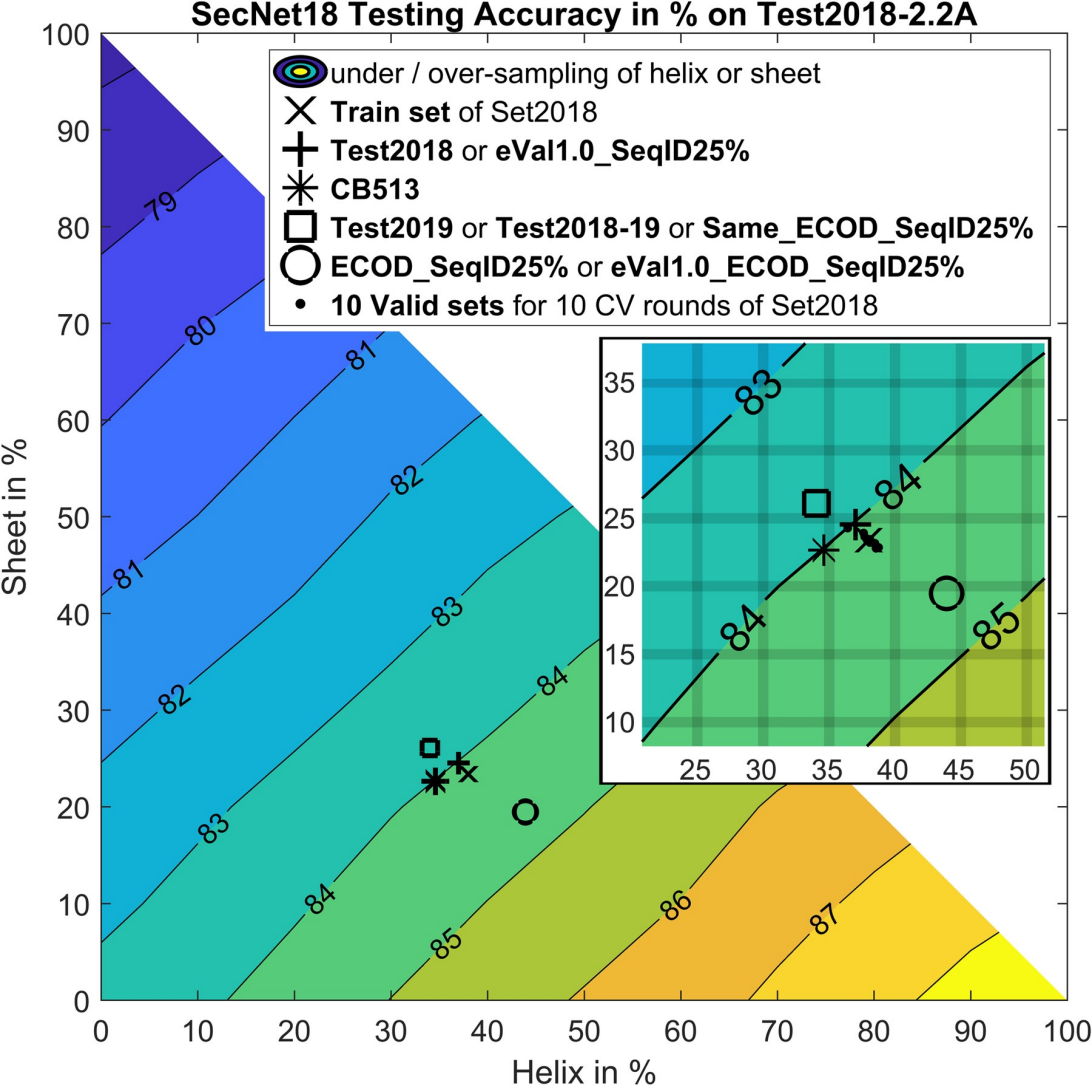
Variation of 3-label *Rule #1* accuracy as a function of proportions of helix (*H%*), sheet (*E%*) and coil (*C%* = 100%–*H%*–*E%*) in *Test2018* test set. The *Rule #1* accuracy of *SecNet* on *Test2018* test set is 84.0% and shown with ‘+’. The contour plot demonstrates how *SecNet* prediction accuracy can be skewed if a test set is enriched or diluted with helix and/or sheet. For example, if the underlying test set has 100% helices, the accuracy rises to 88.7% (bottom right). If it is 100% sheet, the accuracy drops to 77.6% (top left). If it is 100% coil, the accuracy is 83.4% (bottom left). The extrapolated *SecNet* accuracies are indicated for the label proportions of *Set2018* training set with ‘x’, *CB513* test set with ‘*’ and ten validation sets for each cross-validation round of *Set2018* with ten ‘.’. *eVal1*.*0_SeqID25%* has label frequencies almost identical to *Test2018* and shares ‘+’. For the same reason *Test2019*, *Test2018-19* and *Same_ECOD_SeqID25%* share ‘□’ while *ECOD_SeqID25%* and *eVal1*.*0_ECOD_SeqID25%* are shown with ‘o’. The differences in extrapolated accuracies of these sets call for adjustment of the actual accuracies (“Raw values” in Table 9) to the accuracies with the same label proportions (“Adj. to Test2018” in Table 9).

Finally, the accuracy of secondary structure prediction methods for 3 labels depends on the helix, sheet, and coil fractions in the test set. If we oversample *E* or *H*, we can vary the accuracy from 78% to 89% accuracy respectively (Fig 3). As it turns out, our training and test sets and *CB513* are very similar to each other in terms of the 3-label fractions of both *Rule #1* with 37%, 24%, and 39% and *Rule #2* with 34%, 23%, and 43% for helix, sheet, and coil respectively (Fig 3 and Table 3). This should be kept in mind when deriving new test sets.

One further aspect of deriving training and testing data sets for secondary structure prediction (or other structure prediction tasks) is the evolutionary relationships (if any) between any protein in the training/validation set and the testing data set. It is common to use a maximum sequence identity of 25% between proteins in the training/validation sets and the testing set (the sequence identity within each set is a distinct parameter). At this sequence identity, it is likely that there are many pairs of homologous proteins shared by the training/validation and testing data sets. It is possible that *PSI-BLAST* and HMM profiles may be quite similar for two proteins that are homologous but less than 25% identical, and this presents a risk of overtraining. Stricter criteria for separating related proteins are: (1) enforcement of an e-value < 1.0 in a pairwise sequence alignment; (2) association with two different protein super-families according to a structure-based classification; (3) fulfillment of both requirements, each of the three in addition to the < 25% sequence identity by *HHblits* and *Clustal-Omega*. For the pairwise sequence alignment, we used the *PSI-BLAST* profiles derived from Uniref90 database with two rounds exactly as it had been done for *SecNet* features during training. For the structural classification, we used the ECOD database (Evolutionary Classification of Domains) [102], which rigorously groups protein domains into broad classes (the X level, such as all-helical proteins) and then “homology groups” (the H level), which places all homologous domains into single groups. The H level classification includes very remotely related proteins and is performed manually by the Grishin group with extensive expertise in identifying such relationships. Underneath the H level, there is a topology (T) level and protein family (F) level. Some homologous proteins may have additional elements of secondary structure and therefore, somewhat different topologies even though they share a (remote) evolutionary relationship. Some protein chains or entries were missing in ECOD and required extensive processing (refer to *Methods*).

We created four new testing sets as follows. First, from the *Test2018-19* set of 352 test proteins, we removed any protein with a *PSI-BLAST* e-value < 1.0 with any protein in the training/validation set. This set is called *eVal1*.*0_SeqID25%* and has 95 proteins. Second, using ECOD to remove homologous proteins (with the same X.H group domains) of the training/validation set from *Test2018-19*, however, resulted in only 60 proteins. To increase the number of proteins using ECOD, we first selected proteins within the entire PDB of any year having ECOD domains different from the ones in *Set2018* training and validation sets and then enforced the same requirements of the sequence identity, resolution and R-factor as in *Test2018-19*. This set, in *ECOD_SeqID25%*, has 190 proteins. Third, *eVal1*.*0_ECOD_SeqID25%* was formed by applying the e-value < 1.0 requirement between each protein in *ECOD_SeqID25%* and each protein in *Set2018* training and validation sets. Fourth, and finally, we created a positive control testing set (*Same_ECOD_SeqID25%*) of proteins from *Test2018*, *Test2019*, *Test2018-19* that share an ECOD H-level with proteins in the *Set2018* training and validation sets. These should be easier than the first three sets just described. Finally, because secondary structure prediction accuracy can depend on the proportions of helix, sheet, and coil in a test set and these sets indeed demonstrate variation in label frequencies (Fig 3), we adjusted the accuracy on each of these sets by scaling them to the proportions of each secondary structure type (3 labels or 8 labels) as in the Test2018 set.

In Table 9 we present *SecNet* accuracy results on the control set and 3 testing sets with stricter criteria on homology relationships. We include both the raw results and the accuracies adjusted for label content. In the raw results, the more remote testing sets are at most 1.4 points lower in accuracy than the original testing sets (*Test2018*, *Test2019*, *Test2018-2019*). In the results adjusted for label contents, the more remote sets are up to 3 points worse than the original testing sets. This suggests that a criterion of unrelated proteins based on the sequence identity < 25% used in this study and many similar studies is imperfect; a more accurate estimate of the prediction accuracy is achieved by applying the additional criteria. However, the difference is not very large. In the case of the control testing set (*Same_ECOD_SeqID25%*) with each protein sharing at least one *ECOD* domain in the training set, the results are at most 3 points higher than the most remote sets.

**Table 9:** Prediction accuracy of our *SecNet* software trained on *Set2018* and tested on sets with stricter criteria of unrelated proteins.

Test set	Criteria of unrelated proteins			Accuracy				# of proteins in test set
				Adj. to Test2018 label proportions^†^		Raw values		
	Seq.ID <25%	eVal <1.0	Diff. ECOD	8 labels	3 labels, Rule #1	8 labels	3 labels, Rule #1	
Same_ECOD_SeqID25%	+	-	control	74.6	85.1	74.0	84.9	250
Test2019	+	-	-	74.4	84.8	74.0	84.7	220
Test2018-19	+	-	-	74.1	84.7	73.7	84.5	352
**Test2018**	**+**	**-**	**-**	**73.0**	**84.0**	**73.0**	**84.0**	**149**
eVal1.0_SeqID25%	+	+	-	72.3	83.5	72.6	83.5	95
ECOD_SeqID25%	+	-	+	72.0	83.1	73.8	83.9	190
eVal1.0_ECOD_SeqID25%	+	+	+	71.5	82.6	73.1	83.4	72

† Testing sets have varying proportions of 3 and 8 ground-truth labels. Some labels such as alpha helix (H) are the easiest to predict both in 3 and 8 labels while some labels such as beta sheet (E) in 3 labels or bend (S) or bridge (B) in 8 labels are harder to predict. To make the testing sets comparable, we also adjusted their overall accuracies to the same label proportions as in Test2018 set.

### Choices that affect the accuracy of secondary structure prediction

**Fig 4:**
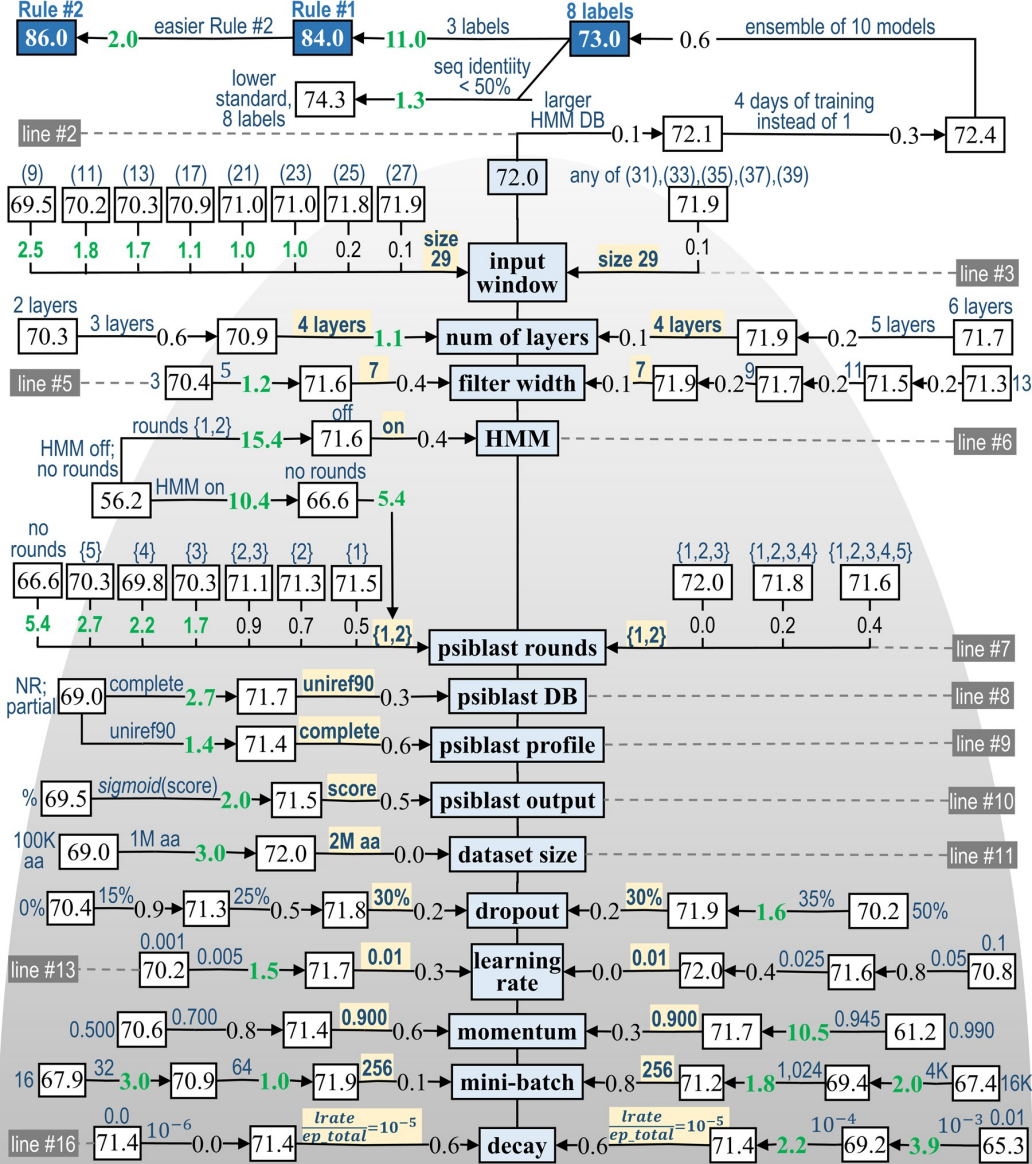
Ablation study of *Test2018* accuracy of *SecNet*. Accuracy is presented as a function of factors (blue boxes in the middle) from 3 groups: 1) NN architecture and complexity (top); 2) input features and databases (center); and 3) hyper-parameters of the training pipeline (bottom). The test accuracies displayed in black uncover unbiased estimates for publication purposes *after* all choices of the final model parameters were made; the validation set was used to make these choices of hyper-parameters using our optimization strategy. *Lines 1–2*: Actions such as “4-day training” or “ensemble of 10 cross-validated models” led to further improvement. *Line 1*: The accuracy increases with switch of 8→3 labels by replacing a harder *Rule #1* to easier *Rule #2* and with a weaker 25%→50% maximal sequence identity between the test and training sets. Each arrow indicates a direction of favorable parameter change and embeds associated accuracy gain. Parameter values are in blue. The optimal parameter values are highlighted in yellow and shown in the middle with smaller and larger values on the left and right. The cumulative effect from multiple changes of the same hyper-parameter is a sum of individual accuracy increments (in black). Stronger effects (≥1 point) are shown in green. The accuracy is a non-linear function of hyper-parameters; an effect from a change of multiple hyper-parameters is not the sum of the accuracy increments associated with the different hyper-parameter changes while the remaining parameters are fixed.

We explored many different ideas and options during the development of *SecNet*. We can divide these choices into three categories: (1) neural network type, architecture, and complexity; (2) input features; and (3) how to perform training of the neural network. After optimizing hyper-parameters (optimization strategy in *Methods*), final training of *SecNet* using the validation set multiple times and probing the *Test2018* set only once to report the final performance (Table 4), we performed an ablation study of the effect of these factors on the 8-label testing accuracy. Some of the necessary CNNs were produced during the development of *SecNet* and some needed to be generated for the ablation study. The results are shown in Fig 4. The first line of the figure lists the three accuracy measures achieved by making various choices shown in the rest of the figure: 86.0% for the 3-label accuracy from *Rule #2* (*H*→*H*; *E*→*E*; all others →*C*), 84.0% for the 3-label accuracy from *Rule #1* (*G*, *H*, *I*→*H*; *B*, *E*→*E*; all others *C*), and 73.0% for the 8-label accuracy. Starting from the middle of the second line, using a “larger HMM DB”, “4 days of training instead of 1”, and “ensemble of 10 models” raise the accuracy from 72.0% to 73.0% in increments of 0.1, 0.3, 0.6 points respectively. A majority of individual accuracy improvements in the figure are a fraction of a percent; it is the cumulative effect of many choices that makes a significant improvement in accuracy.

Each line below the second line ablates one of the factors, such as a feature, algorithmic, or training choices described in a blue rectangular box; the highest testing accuracy of 72.0% is shown in the middle of the figure achieved with a set of the locally optimal parameters found during the optimization (refer to *Methods*) using the validation set. The accuracy as a function of the hyper-parameters has numerous local maxima. Our optimization strategy did not lead to the global maximum; at best, it found the maximum with the highest accuracy among a limited number of local maxima tested. Non-optimal parameters are ordered on the left and right with lower and higher values compared to the optimal values respectively. The accuracy as a function of hyper-parameters is highly non-linear; its change is *not* a sum of individual accuracy changes associated with individual hyper-parameter variations. Many subsets of hyper-parameters such as (learning rate, momentum, decay) or (network depth, convolutional width) exhibit a strong interaction and have been optimized together. In the ablation study we only demonstrate ablation of a single factor away from the local optimum while keeping the remaining hyper-parameters fixed.

The first category of choices involves the architecture of the neural network and its complexity. For example, secondary structure formation is influenced by short, middle, and long-range inter-residue interactions. We were interested to see how the accuracy relates to the size of the sequence “input window” (line 3 in Fig 4) centered on a residue subject to prediction. To investigate this, we varied the input window size, and observed increasing accuracy from 69.5 to 71.9% for a window of 9 to 27 (left), the highest 72.0% for the optimal window of 29 (middle) and subsequent degradation to 71.9% for a window of 31 to 39 (right). The severe degradation of the accuracy (1.7–2.5 points) was observed for 9–13 amino-acid-wide windows, moderate reduction (0.1–1.1 points) was for 17–27 wide windows and very minor decrease (0.1 points) was for 31–39 wide windows.

Another important factor in secondary structure prediction accuracy is the kind of neural network, convolutional filter width, and the number of layers. The 4-layer NN is more accurate by 1.7 and 1.1 points than 3-layer and 2-layer NNs and by 0.1 and 0.3 points than 5-layer and 6-layer NNs (line 4). Wang et al. made a similar observation of accuracy saturation at 5–7 layers [75]. The convolutional filter width of 7 is the most accurate; there is slight accuracy degradation of 0.1 and 0.4 points with wider and narrower filter widths of 9 and 5 respectively. Further expansion of the width by 2 drops accuracy by additional 0.2 points each time while narrowing of the filter width from 5 to 3 severely decreases accuracy by an additional 1.2 points. The filter narrowing below 7 is more damaging than expansion because 3_10_-, α- and π- helices form hydrogen bonds between amino-acid residues with 3, 4 and 5 residues apart, and an average length of beta strands is about 6 [103].

We also tested a more complex residual neural network with 20–40 layers and a 21–51 amino-acid window size, and observed the same or worse accuracy than *SecNet*’s traditional CNN with 4 layers (Fig A in S1 File). We underline that the residual neural networks did not fail and produced the same accuracy with several different architectures; the residual neural networks were abandoned due to their higher complexity both during training and final application. Coupled with the results from Table 5, we conclude that a CNN is adequate for secondary structure prediction, despite availability of newer and more complex neural architectures.

The second category of choices consists of the features used as NN inputs. The use of sequence profiles, as demonstrated by many programs over the last 30 years, has a strong positive impact on accuracy (line 6). If both the *PSI-BLAST* and *HMM* features are disabled, the accuracy drops from 72.0% to only 56.2% (“HMM off; no rounds”), contributing to the largest observed impact of all factors we explored in the ablation study. When either *HMM* or *PSI-BLAST* are individually enabled, the accuracy jumps to 66.6% and 71.6% respectively.

A choice of database for the *PSI-BLAST* searches is not that critical. *Uniref90* is only 0.3 points better than the *NR* non-redundant sequence database from NCBI (line 8), probably because for some targets *NR* produces too many nearly identical hits, and subsequently skews the *PSI-BLAST* profile too much in their favor. There is a *PSI-BLAST* command-line argument that determines how many of the top hits are included in a profile; if the profile is limited to the top 15 hits, the accuracy suffers by a hefty 2.7 points for *NR* (“NR; partial” vs. *NR*’s “complete” in line 8) and only 0.6 points for *Uniref90* (line 9). *PSI-BLAST* produces profiles both in log-odds form and amino-acid percentages. The log-odds form (“score”) is better than the percentage values by 2.5 points (line 10). It is common to use a sigmoid function to transform a feature into a 0 to 1 range. The log-odds score is also better than their sigmoid function values (“*sigmoid*(score)”)—by 0.5 points.

*PSI-BLAST* profiles generated after a certain number of rounds have a significant effect (line 7). Profiles obtained from the third, fourth, and fifth rounds contain homologues that are very distant from a target sequence and therefore may contain significant differences in secondary structure, which seem to adversely affect the accuracy. The accuracy of neural networks trained on profiles after the third, fourth, and fifth rounds alone drops respectively by 1.7, 2.2, and 2.7 percentage points relative to the best option which includes both profiles after the first and second rounds. This best option is higher by 0.5 and 0.7 points than using the first or second round profiles alone. We observe a steady but modest degradation of accuracy by 0.0 or 0.2 or 0.4 points when a combination of rounds 1–3, 1–4, or 1–5 are used. A possible explanation is that the additional rounds decrease the data/parameter ratio. The round 2–3 combination is worse by 0.9 points than the round 1–2 combination.

The third category of choices involves the training options. A difference in training data set size of 1 or 2 million amino-acids has no effect, but a 10-fold training data set reduction to 100 thousand amino acids pushes accuracy down by 3 percentage points (line 11). For regularization, we chose the dropout technique by adding a dropout layer at each NN layer (including the input and dense layers) which effectively randomly sets 30% of the input features and model parameters to zero during each minimal training iteration with a subsequent restoration (line 12). This approach results in 1.6 points higher accuracy compared to training without any dropout. The 30% dropout is optimal; the accuracy drops when dropout is either decreased or increased from 30%. CNN is relatively insensitive to small changes in learning rate from 0.01 and momentum from 0.900. However, larger deviations from these values result in significant accuracy losses; for example, the accuracy loses 0.3–1.8 points and 0.4–1.2 points when the learning rate is 0.001–0.005 and 0.05–0.1 respectively (line 13). A momentum of 0.500 instead of 0.900 results in a 1.4 points drop while a value of 0.990 reduces accuracy by 10.8 points (line 14).

Each weight update in training our network involves calculating derivatives of the loss function for a batch of data, called a “mini-batch.” If the mini-batch is too small, then the calculated derivatives will be too noisy; mini-batch sizes of 16–64 instead of 256 amino acids produced losses of 0.1–4.1 points in accuracy. A mini-batch size that is too large may produce too stable loss function gradient and may result in premature convergence of the model to a less optimal set of parameters; mini-batch sizes from 1,024 to 16,384 resulted in a loss of 0.8–4.6 points (line 15).

In order to find a better optimum during the training and especially toward its end, it is critical to reduce the size of gradient-descent steps by decreasing the current learning rate (“decay”). A formula-based decay of “learning rate / epoch total” which is equal to 10^−5^ with the optimal learning rate of 0.01 and 650 epochs for 4 days, is better by 0.6 points than no decay (0.0) or smaller decay of 10^−6^; it is also better by 0.6, 2.8 and 6.7 points than a larger decay of 10^−4^ or 10^−3^ or 0.01 respectively (line 16). Finally, if we permute proteins in the whole data set minus the unchanged testing set and split them into different training and validation sets *many* times, the validation accuracy becomes overestimated by 2.3 points due to easier targets in the favorable random validation set; however, we observe no improvement in the testing accuracy. Linear or non-linear random sampling of central residues either within proteins or the complete training set had no effect on the validation or testing accuracies. Other training options were described earlier (lines 1–2), resulting in a final accuracy of 73.0%.

We conclude that the best results are achieved with good choices for input features, sequence databases and arguments, and the training pipeline and regularization techniques. It appears that the NN type, architecture and complexity, and the associated number of training parameters are not as critical as long as the NN is not too simple.

### Practicality of secondary structure prediction: Which and how many labels

Most existing secondary structure prediction programs have been trained and tested on either 8-label or 3-label data sets. In most cases, the 8 *DSSP* labels are reduced to 3 by treating 3_10_ helices (“G” in *DSSP*) and π helices (“I” in *DSSP*) as *H*, and single-residue beta strands (beta bridges or “B” in *DSSP*) as *E*; the other labels (“C” or coil, “T” or turns, “S” or bends) are reduced to *C*. We wondered how label sets with more than 3 but fewer than 8 labels would behave and how these might be developed.

To investigate this, we calculated the confusion matrix for 8-label *SecNet* (Table 2) and both the column and row-normalized versions of the confusion matrix. The column-normalized table (Table 2, middle) provides true positive rates (“recalls”) along the main diagonal (TPR = TP/(TP+FN)) and false negative rates (FNR = FN/(TP+FN)) off the diagonal; the row-normalized table (Table 2, bottom) reports positive predictive values (“precisions”) along the diagonal (PPV = TP/(TP+FP)) and false discovery rates off the diagonal (FDR = FP/(TP+FP)).

The column-normalized confusion matrix shows that only labels *H*, *E*, *C*, and *T* have true positive rates over 50%. The TPR for beta bridges (“B”) is only 2.8%. Experimental *G* and *I* are predicted as *H* more often than their true labels. Bends (“S”) are predicted as *C* almost twice as often as they are predicted as *S*. The row-normalized confusion matrix shows that all 8 labels have positive predictive values over 50%, but they are more than 60% for only *H* (85%), *E* (80%), and *I* (100%). *I* labels, however, are only 0.05% of the true labels and 0.01% of the predictions (Table 2, top).

From a practical point of view, predicted secondary structure labels are useful if they are accurate (high PPV and TPR), commonly observed, and lead to effective sampling strategies in tertiary structure prediction, which might be performed though the use of fragments or from dihedral angle distributions. From this point of view, the *S* and *B* labels are not very useful. The *S* label indicates a “bend” when the angle Cα(i-2)–Cα(i)–Cα(i+2) is less than 110° and residue *i* is not *H*, *B*, *E*, *G*, *I*, or *T* [2]. The *B* label indicates a beta bridge, which is simply a one-residue backbone-backbone hydrogen bond, resembling a one-amino-acid beta-sheet strand. Since *S* and *B* have low TPR values and are impractical to sample effectively, it makes sense to convert them to the catch-all label, *C*, and sample them from Ramachandran distributions generated from residues not in helices or sheets [104]. Similarly, the *I* label is so rare that it may be converted to *H*, since about 75% of the time it occurs as the first or last turn in an alpha helix. Thus, after these conversions, we are left with *H*, *E*, *C*, *T*, and *G*.

We trained and tested a 5-label version of *SecNet* (*H*, *E*, *C*, *G*, *T*), and achieved an accuracy rate of 78.5% (Table 10), compared to 84.0% for the *Rule #1* 3-label predictions, 86.0% for the *Rule #2* 3-label predictions, and 73.0% for 8-label predictions. The TPR values for the 5-label rules for *H*, *C*, *E*, *T*, and *G* are 94%, 78%, 84%, 52%, and 34% respectively. The PPV values are 89%, 73%, 84%, 69%, and 55%.

**Table 10:** 5-label practical alphabet: *H*, *C*, *E*, *T*, and *G*: (1) true positive rates (recalls) and false negative rates and (2) positive predictive values (precisions) and false discovery rates of *SecNet*, (3) TPRs and FNRs for three subclasses of *G*: **(a)** *G* abut *HH…H*, **(b)** isolated *GGG* in loops and **(c)** isolated *GGGG+* in loops.

**Accuracy**			**True label**						
**78.5%** Column normalized table			**H**	**C**		**E**	**T**		**any G**
			100	100		100	100		100
**Predicted label**		**H**	**94.0**	4.9		1.2	14.9		22.2
		**C**	3.0	**78.1**		14.2	26.7		27.9
		**E**	0.6	10.7		**83.6**	2.3		3.4
		**T**	1.4	5.3		0.8	**52.3**		12.4
		**G**	0.9	1.0		0.2	3.7		**34.2**
**Diagonal has TPRs** Elsewhere FNRs									
Row normalized table			**True label**						
			**H**	**C**		**E**	**T**		**any G**
**Predicted label**	**H**	100	**88.6**	3.9		0.7	4.5		2.3
	**C**	100	3.3	**73.4**		10.3	9.6		3.4
	**E**	100	0.9	13.8		**83.6**	1.1		0.6
	**T**	100	5.7	18.2		2.1	**68.5**		5.5
	**G**	100	13.3	12.5		1.9	17.6		**54.7**
**Diagonal has PPVs** Elsewhere FDRs									
Column normalized table			**True label**						
			**G abut H**		**GGG**			**GGGG+**	
			20% any G		55% any G			25% any G	
			100		100			100	
**Predicted label**		**H**	41.8		12.4			27.6	
		**C**	12.7		33.7			27.4	
		**E**	0.6		4.1			4.0	
		**T**	9.9		14.5			10.0	
		**G**	**34.9**		**35.3**			**31.0**	
**1 TPR** + 4 FNRs **for subclasses of G**									

The 5 practical labels are defined as: (*S*, *B*) → (*C*) and (*I*) → (*H*). *Bottom*: Same 5-label alphabet. The *G* recalls and FNRs are shown for 3 subclasses of G where *any* true *G*‘s (*G* column in the *Middle* section) are assigned into: (a) *GG…G* abut *HH…H*, (b) isolated stretch of 3 *G*’s in loops and (c) isolated stretch of 4 or more *G*’s in loops as well. The dominant FNR of 33.7% for *C* in the prevailing *GGG* subclass of 55% and almost equal FNRs for *H* and *C* of 27.6% and 27.4% in the 25% *GGGG+* subclass outweigh the dominant *FNR* for *H* of 41.8% in the minority *G*-abut-*H* subclass of only 20%.

The label *G* (3_10_ helices) remains problematic in the 5-label scheme with false negative rate of 28%, 22%, and 12% for *C*, *H*, and *T* compared to a 34% true positive rate for *G*. In a recent study on beta turns [105], we divided 3_10_ helices into three categories: 3_10_ helices that abut alpha helices (20% of *G*), isolated 3-residue 3_10_ helices (the most common length by far) in loop regions and not abutting alpha helices (55% of *G*), and isolated 3_10_ helices of length 4 or more (25% of *G*). 3_10_ helices that abut alpha helices might be viewed as a distortion of the alpha helix and therefore might be more similar to *H* labels. Isolated 3_10_ helices of length 3 strongly resemble the succession of two Type I beta turns (with distorted backbone dihedral angles for the first turn compared to other Type I turns) [105].

We analyzed the TPR and FNR values for these three categories of residues in 3_10_ helices from the 5-label predictions (bottom of Table 10). With *SecRes* trained on a 5-letter scheme, *G* residue stretches that abut alpha helices have a 35% true positive rate and false negative rates of 42%, 13%, and 10% for *H*, *C*, and *T* respectively. Isolated *GGG* segments in loops have a 35% true positive rate and false negative rates of 12%, 34%, and 15% for *H*, *C*, and *T* respectively. Finally, isolated 3_10_ helices of length 4 or longer produce a TPR of 31% and FNR values of 28%, 27%, and 10% for *H*, *C*, and *T* respectively. With these results in hand we derive a 4-letter scheme (*H*, *E*, *C*, *T*) by converting all *G* to *C*, since 80% of *G* residues are isolated 3_10_ helices of length 3 or longer. Since the majority of residues in 3_10_ helices come from isolated length-3 helices which are very similar to beta turns, retaining the prediction of turns (label *T*) may enable the sampling of these structures in loop regions. As before, *B* and *S* are converted to *C*, and *I* is converted to *H*.

The accuracy for this 4-label practical scheme is 79.9%; the column- and row-normalized confusion matrices for this scheme are provided in Table 11. It achieves very high TPRs of 94%, 79%, and 83% and PPVs of 90%, 75%, and 85% for *H*, *C* and *E*; it achieves satisfactory TPR of 50% and PPV of 71% for *T*. If simpler, practical labels are required, *T* may be replaced with *C* as the best candidate both structurally and in terms of the highest false negative rate of 33% and highest false discovery rate of 22%. The final accuracy for this new 3-label prediction scheme is 86.0%; its TPR (recall), FNR, PPV (precision) and FNR are in Table 12.

**Table 11:** 4-label practical alphabet: *H*, *C*, *E*, and *T*: (1) true positive rates (recalls) and false negative rates and (2) positive predictive values (precisions) and false discovery rates of *SecNet*.

**Accuracy**			**True label**						
**79.9%** Column normalized table			**H**		**C**	**E**		**T**	
			100		100	100		100	
**Pred. label**		**H**	**93.7**		6.5	1.3		14.4	
		**C**	4.4		**79.3**	15.6		33.3	
		**E**	0.5		9	**82.5**		2.1	
		**T**	1.4		5.2	0.6		**50.2**	
**Diagonal has TPRs** Elsewhere FNRs									
Row normalized table			**True label**						
			**H**	**C**			**E**		**T**
**Pred. label**	**H**	100	**88.9**	5.9			0.8		4.4
	**C**	100	4.4	**74.9**			10.1		10.6
	**E**	100	0.8	13.5			**84.7**		1.1
	**T**	100	6.1	21.6			1.7		**70.6**
**Diagonal has PPVs** Elsewhere FDRs									

The 4-label alphabet is defined as: (*S*, *B*, *G*) → (*C*) and (*I*) → (*H*).

**Table 12:** Final 3-label alphabet: *H*, *C*, and *E*: (1) confusion matrix, (2) TPR and TNR and (3) PPV and FDR of *SecNet*.

Confusion matrix of 37,620 test labels					
**Accuracy**		**Pred. freq.**	**True label**		
**86.0%**			**C**	**H**	**E**
**True freq.**		100%	43.4	33.3	23.3
**Predicted label**	**C**	44.3	**37.1**	2.8	4.4
	**H**	34.0	3.5	**30.3**	0.3
	**E**	21.7	2.9	0.1	**18.7**
Column normalized table			**True label**		
			**C**	**H**	**E**
			100	100	100
**Predicted label**		**C**	**85.3**	8.6	18.9
		**H**	8.0	**91.1**	1.1
		**E**	6.7	0.3	**80.0**
**Diagonal has TPRs** Elsewhere FNRs					
Row normalized table			**True label**		
			**C**	**H**	**E**
**Predicted label**	**C**	100	**83.6**	6.4	9.9
	**H**	100	10.2	**89.0**	0.8
	**E**	100	13.4	0.5	**86.1**
**Diagonal has PPVs** Elsewhere FDRs					

The final 3-label rule is defined as: (*S*, *B*, *G*, *T*) → (*C*) and (*I*) → (*H*). The *Rule #2* requires *I* → *C*, in contrast to this rule, which converts *I* → *H*; otherwise they are identical. Since the *I* frequency within the test set is negligible 0.05%, there is no difference in the final accuracies of the two alphabets.

To summarize, we suggest new 5- and 4-label schemes as follows and abandon new 3 labels for the reasons outlined below:

- 5 new labels: (*E*) → *E*, (*H*, *I*) → *H*, (*C*, *S*, *B*) → *C*, (*G*) → *G*, (*T*) → *T* (Table 10)
- 4 new labels: (*E*) → *E*, (*H*, *I*) → *H*, (*C*, *S*, *B*, *G*) → *C*, (*T*) → *T* (Table 11)
- 3 new labels (abandoned): (*E*) → *E*, (*H*, *I*) → *H*, (*C*, *S*, *B*, *G*, *T*) → *C* (Table 12)

This last 3-label alphabet is neither *Rule #1* nor *Rule #2* 3-label alphabets found in the literature. It very closely resembles *Rule #2* of the 3-label alphabet except for a different conversion of *I* label (0.017% of all *Set2018* residues); due to the negligible frequency of *I*, there is no observable difference in training nor the overall accuracy (86.0%) between this rule and *Rule #2*. Therefore, we abandon this new 3-label scheme and keep the traditional Rule #2 instead. In Table 13 we summarize existing and new definitions and compare accuracy of all six alphabets.

**Table 13:** Definitions of different label alphabets and associated *SecNet* accuracies on *Test2018* test set.

Alphabet	Definition	Labels	Accur.	Diff.
8 labels	8 original DSSP labels unchanged (*C, S, B, T, I, G, H, E*)	*H, E, C, T, G, S, B, I*	73.0	0.0
5 labels new, kept	(*C, S, B*) → *C*, (*H, I*) → *H*, (*E*) → *E*, (*T*) → *T*, (*G*) → *G*	*H, E, C, T, G*	78.5	+5.5
4 labels new, kept	(*C, S, B, G*) → *C*, (*H, I*) → *H*, (*E*) → *E*, (*T*) → *T*	*H, E, C, T*	79.9	+6.9
Rule #1 3 labels	(*C, S, T*) → *C*, (*H, I, G*) → *H*, (*E, B*) → *E*	*H, E, C*	84.0	+11.0
Rule #2 3 labels	(*C, S, B, T, I, G*) → *C*, (*H*) → *H*, (*E*) → *E*	*H, E, C*	86.0	+12.0
3 labels new, discarded	(*C, S, B, G, T*) → *C*, (*H, I*) → *H*, (*E*) → *E*	*H, E, C*	86.0	+12.0

### Usage of our secondary-structure prediction software

Our secondary structure prediction software, *SecNet*, is free, open-source, and cross-platform. It is written in *Python* 3; a 20KB online installer is downloadable from dunbrack.fccc.edu/ss and github.com/sh-maxim/ss. When the *SecNet* installer is launched, it prompts for a single destination directory, self-configures, downloads and installs a required version of embedded *Python*, detects and installs the required (but missing) *Python* libraries such as *Numpy*, *Keras*, and *Theano*, downloads and installs necessary third-party software and databases. The uninstallation is simple as deleting a kit directory. The prediction mode of our application requires an average desktop with 2–4 CPU cores and 2–4 GB of RAM; during execution, it consumes 1-2 GB of RAM. For faster multi-threaded execution, the software detects how many CPU cores are available and uses all of them in the third-party sequence alignment software and running NN code as well unless fewer cores are assigned with an optional argument.

A user does not need to generate any input features manually; *SecNet* automatically prepares all required input features for a target protein sequence by running included third-party software with preprocessed sequence databases. For an average protein of 200–300 residues it takes about 5–10 minutes to prepare input features, several seconds to load predictive models from a storage device into memory, and several seconds to run an ensemble of 10 NN models to perform secondary structure prediction. The usage is very simple with intuitive command-line arguments such as—label [3 or 4 or 5 or 8 or all] and—rule1 or—rule2; the full instructions and options are available at the above online resources, with “—help” flag and also included in a readme file:

*secnet -i example.seq*—*label 8 -o example.ss8*
*secnet -input example.fasta*—*rule1*—*label 3*—*output example.rule1ss3*
*secnet -i example.seq -l 4 -o example.ss4*—*cpu 3*

## Discussion

We have developed *SecNet*, a simple traditional four-layer convolutional neural network for predicting protein secondary structure purely from sequence. We constructed new training, validation, and testing sets such that the test set consists of protein structures determined in 2018 that had no more than 25% sequence identity with any structure released in the PDB prior to January 1, 2018. This enabled fair and unbiased comparison of our method with two programs, *DeepCNF* and *eCRRNN*, which were trained on data released prior to 2018 and which had the highest reported accuracies on *CB513*, a widely used test set in secondary structure prediction studies. *SecNet* achieved accuracy of 73.0% on 8-label predictions, 84.0%, on *Rule #1* 3-label predictions, and 86% on *Rule #2* 3-label predictions on *Test2018*. It outperforms *DeepCNF* and *eCRRNN* by 2.0–3.4 percentage points on our *Test2018* set and *CB513*, and other methods by 1.7–8.8 points on the *CB513* test set. While this is a small improvement in accuracy, it is significant in light of progress over the last 20 years—the accuracy of *PSIPRED*, published in 1999, on *CB513* was 79.2% for 3-label *Rule #1* predictions.

The 25% sequence identity cutoff between training and testing sets in secondary structure prediction is very common. We performed an additional check that this cutoff did not result in overtraining (by having homologous proteins in the training and testing sets with < 25% sequence identity but with similar *PSI-BLAST* profiles). We developed an alternate testing set by applying the definitions of homologous domains from the ECOD database (Evolutionary Classification of Domains). This test set of 190 proteins had no domain at the ECOD H level in common with any protein in the training/validation set. *SecNet* tested on this new set had higher 8-label accuracy and only 0.1 point lower (83.9% vs 84.0%) on 3-label accuracy than it did on *Test2018*. The results with a *PSI-BLAST* e-value cutoff of < 1.0 were similar (Table 9). These sets may in fact be slightly easier than *Test2018*. If we adjust them for their proportions of different secondary structure types to be identical with *Test2018*, their accuracy drops by one percentage point.

In the development of *SecNet*, we tried a variety of options for feature calculation, network structure, and training hyper-parameters. After selecting the best options with the validation set, we used the test set only once to estimate the accuracy. We then performed a retrospective ablation study, using the test set to calculate accuracy on the various networks produced during the earlier training and validation studies. The results not surprisingly indicate that the most accurate model is the result of many decisions each of which contributes toward the accuracy. The most interesting results of the ablation study were that window sizes larger than 29 residues did not increase the accuracy, and that *PSI-BLAST* profiles are more useful than HMMs in predicting secondary structure, even though the information in HMMs is inherently richer.

From our experience in developing the training, validation, and test set, we developed a short list of recommendations that might be considered in future efforts to avoid overtraining and to enable comparison of different programs:

### 1) Generating a list of chains for training, validation, and test sets

A majority of prediction methods enforce a maximal pairwise sequence identity of 25% between training, validation, and/or test sets as well within the test set. But how the sequence identities are calculated can matter, and this is not always described in sufficient detail to ensure reproducibility. A few methods either do not list a threshold [77, 80] or use a higher value [81, 92], which may lead to a higher accuracy that would not be sustained on proteins unrelated to the training and testing sets [82]. For example, we observed that if our model is trained and tested on data sets with 50% sequence identity instead of 25%, it has 1.3 points higher accuracy (top of Fig 4). This issue has been raised several times by different groups [34, 46, 90, 91]. In this work, we developed a rigorous protocol that relies on two passes of two different sequence alignment software programs (*HHblits* and *Clustal-Omega*) to enforce 25% identity within our data sets.

An unforeseen violation of a sequence-identity threshold may occur when a method relies on predictions from third-party software as input features when the third-party software was trained on protein structures that are more than 25% identical to sequences in the test set of the new method. This may occur for instance, if a method uses features such as predicted contacts or solvent exposure from other programs trained on sequences related to those in the test set. This can be avoided if the test set for the secondary structure prediction method contains sequences of structures determined that are less than 25% identical to the training sets of all predicted input features. This can be hard to determine, since not all training sets are defined by authors of feature prediction programs or there is a set of nested programs with each trained on its own training set. To avoid such a situation, we should enforce the pairwise identity threshold between every new structure published after the third-party software release and those before it. It is necessary to compare all sequences before and after the required date, regardless of resolution, experimental method, or structure quality. These filters can be applied to the training, validation, and test data later.

Another consideration on which chains to include in the training, validation, and test sets is their secondary structure content. Alpha helices are easier to predict and a test set enriched with them is biased to have higher accuracy (Fig 3); therefore, data sets should have secondary structure labels representative of the general PDB population.

### 2) Sequences and labels for the training, validation, and test sets

Secondary structure cannot be assigned to amino acids with missing coordinates caused by unresolved electron density, and such amino acids are ignored by *DSSP*. A test set should consist of full sequences, and the labels therefore need to include a designation for residues missing from the coordinates. By contrast, the popular *CB513* test set does not include residues with missing coordinates. Information on disordered residues mapped to the sequence is provided in the *mmCIF* format of files from the PDB, or from the file “*ss_dis*.*txt*” available from the PDB (https://www.rcsb.org/pages/download/http#ss).

Previously secondary structure prediction methods are mostly trained to predict three labels: helix (*H*), sheet (*E*), and coil (*C*). In the early days, ground-truth labels came from secondary structure assigned by authors of deposited PDB structures. With emergence of a widely-adopted *DSSP* program in 1983, the 8 secondary-structure labels (*H*, *B*, *E*, *G*, *I*, *T*, *S*, *C*) assigned by this software have been converted into 3 labels (*H*, *E*, *C*); the sets of 8 and 3 labels are used separately for development of prediction methods. With a lack of standardization, methods report accuracies based on either *Rule #1*: (*H*, *G*, *I*) → *H*, (*E*, *B*) → *E*, (other 4 labels) → *C* or *Rule #2*: (*H*) → *H*, (*E*) → *E*, (other 6 labels) → *C*. The 3-label *Rule #2* is easier for prediction than *Rule #1* by 2.0 percentage points (top of Fig 4). Some authors did not report which rule was used [77, 78, 81, 83]; some continue to use the easier *Rule #2* and compare their results to previous methods that used the harder *Rule #1* [80, 93]. Which rule is used should be clearly stated in a publication to avoid ambiguity.

Label sets greater in size than 3 but less than 8 may be useful in some contexts. We demonstrated that the *B* (beta bridge) and *S* (bend) labels have very little sequence signal, and since they may be difficult to sample anyway they can productively be converted to the generic *C* label. The *I* label (pi helices) is very rare, and may be converted to *H*, producing a 5-label rule: (*H*, *I*) → *H*; (*B*, *S*, *C*) → *C*; *E; T*; *G*. Residues in 3_10_ helices are also difficult to predict. We showed that those 3_10_ helix residues immediately adjacent to alpha helices have similar amino-acid preferences to alpha helices. Those isolated within coil regions, which are commonly of length 3, are mostly predicted as coil. However, these residues outnumber those adjacent to alpha helices, and *G* can therefore be productively converted to *C*. Therefore, we define a 4-label rule as: (*H*, *I*) → *H*, (*B*, *S*, *C*, *G*) → *C; E; T*. Finally, we define a slightly modified version of *Rule #2*: (*H*, *I*) → *H*; (*B*, *S*, *C*, *T*) → *C; E*.

### 3) Separation of training, validation, and test sets

Some methods do not designate a separate validation set, and make design decisions based on repeated use of either the training set or a single test set. If a training set is used for these purposes, the produced model is over-trained and training accuracy will not propagate to the test set accuracy. For example, the accuracy on our validation set is 2.0 points and 2.5 points higher than that on the test sets for 3-label and 8-label predictions respectively. A validation set should be split from the training set and used for model selection, with the test set only used at the end to produce final accuracy estimations [106].

### 4) Distributing the data sets, including sequences and labels

The availability of reliable data sets is critical for reproducibility and fair benchmarking. For many publications, these sets are not readily available; contacting authors is time-consuming and often unproductive, and the needed information is not always still available. Data sets that can be reused by other groups to test new methods need to contain not only the PDB codes and chain identifiers but also the full sequences and label strings. The PDB can occasionally change a sequence and coordinates if an entry is updated. If the 8-label *DSSP* codes are converted to a smaller set, then providing the transformed labels is valuable to retain consistency. Complete data sets should be uploaded to at least two publicly available data sharing systems, for example to journal or institutional websites or *Github* or *Dropbox* or *Sourceforge*. A journal may not always provide adequate storage for bulky data sets.

## Methods

### Protocol for preparation of 2018 data sets

A complete list of PDB entries was obtained from PISCES [27] server on November 12, 2018. The entry list was divided into *Before18*, which consists of entries released prior to January 1, 2018, and *After18* which consists of those released on or after the same date (Fig 1). Chain sequences were obtained from PISCES. Identical sequences were removed from *Before18* and *After18*, and sequences identical to any sequence in *Before18* were removed from *After18*. Each identical sequence was linked with a complete list of its protein structures of varying quality.

We used the output of two sequence-alignment programs, *HHblits* and *Clustal-Omega*, to calculate pairwise sequence identities within and between the sequences in the *Before18* and *After18* sets. According to *HHblits*, a sequence identity is the number of identical amino-acid residues divided by the shortest protein sequence in a pairwise alignment. *Clustal-Omega* returns an identity matrix with “—distmat-out = filename—percent-id” flags. According to *Clustal-Omega*, a sequence identity is the number of amino-acid identities in the alignment divided by the number of residues compared (gap positions are excluded). The *HHblits* pairwise sequence identities were calculated for use in the PISCES server. Any item in *After18* was removed if it had more than 25% sequence identity to any item in *Before18* calculated with either *HHblits* or *Clustal-Omega*. We underline that *Before18* includes PDB sequences of any experiment or resolution to eliminate any test sequence too similar even to a low-quality pre-2018 sequence that could have been included in training of previous methods. Usage of two programs helps if either one fails to detect similarity.

We applied several filters to the structures and sequences in *Before18* and *After18* before applying a pairwise sequence identity filter within each set. We removed structures having resolution worse than 2.2 Å, or free R-factor greater than 0.25 (if free-R factor was not available, we used the R-factor+0.05) [94]. We excluded chains of length less than 40 residues and Cα-atom-only models. Next, we enforced 25% pairwise identity in each set based on the sequence identities obtained from *HHblits* and *Clustal-Omega*. We removed any sequences from *Before18* that was more than 25% identical with any sequence in *CB513* so that we could use *Set2018* to train *SecNet* and use *CB513* as an additional test set (*Test2018* already cannot have sequences more than 25% identical to the structures in *CB513* that were determined before the year 2000). For identical sequences, we selected a protein chain from a crystal structure having the highest resolution, followed by the best R-factor, and then the PDB chain having the largest number of coordinates.

This procedure produced the *Test2018* test set (once the quality and sequence identity filters were applied to *After18*), which contains sequences that are not more than 25% similar to *any* chain in a PDB entry released before Jan 1, 2018. Similarly, the *Set2018* training and validation sets were created from a 9 to 1 random split of the chains in *Before18* after the quality and sequence identity filters were applied. We note that complete chain sequences are stored (not just the ones with coordinates) in our sets. We store original one-letter sequences using the standard representation where a non-standard amino acid has a single-letter code of the closest analog among 20 standard amino acids if it exists; otherwise “X” is used.

### Generation of Test2019 and Test2018-19 data sets

Since *Test2018* only includes proteins released within about a 10-month range (01/01/18-11/12/18), we created two additional data sets, *Test2019* and *Test2018-19*, corresponding to 11/13/18-12/31/19 and 01/01/18-12/31/19 date ranges which cover 14 months and 24 months respectively. We carried out the same protocol shown above for *Test2018*.

### ECOD database, processing and test on two related proteins

The ECOD database, (file: “ecod.develop262.domains.txt”, version: “develop262”, release date: 01/31/2020) was downloaded from http://prodata.swmed.edu/ecod/complete/distribution. For any protein under consideration for our training or testing sets:

1. The ECOD database is queried with PDB_ID + chain_ID.
2. If a record is found, assigned (if any) X.H group intra-chain or inter-chain domains are saved for the protein (some domains extend across two different chains, e.g. trypsin).
3. If such record does not exist in the database, the database is queried with PDB_ID + chain_ID of a protein chain having the same sequence and same PDB_ID.
4. If such protein chain does not exist or such record is not found, the database is queried with PDB_ID + chain_ID of a protein chain having again the same sequence but a different PDB_ID.
5. If such protein chain does not exist or such record is not found, an HTTP request for PDB_ID is made with http://prodata.swmed.edu/ecod/complete/search?kw=PDB_ID and parsed to find whether the ECOD team has already processed the PDB entry either automatically or manually.

To test for a relationship between two proteins, if the sets of saved X.H group domains of the first and second proteins share at least one domain, the proteins are considered related.

### Input labels and features

The 8 secondary-structure labels assigned with *DSSP* of 3.0.5 version (released by CMBI on Oct 11, 2018) were obtained from the file, “*ss_dis*.*txt*” which has records for the entire PDB and is available for download from PDB. This file also contains strings that indicate the ordered and disordered residues in the sequence. The *DSSP* and disorder strings were combined, with spaces in the *DSSP* string replaced with “C” when the disorder string indicated an ordered residue (“-“) and “X” when the disorder string indicated a disordered residue (“X”). For instance, for PDB entry 1A64 chain A, this file contains:

>1A64:A:sequence

RDSGTVWGALGHGINLNIPNFQMTDDIDEVRWERGSTLVAEFKRKPFLKSGAFEILANGDLKIKNLTRDDSGTYN

VTVYSTNGTRILDKALDLRILE

>1A64:A:secstr

EEEEETT EEE TT TTEEEEEEEETTEEEEEEESS EESSTTEEE TTS EEESS GGG EEEE

EEEEETTS EEEEEEEEEEEE

>1A64:A:disorder

XXX - - - - - - - - - - - - - - - - - - - - - - - - - - - - - - - - - - - - 

 - - - - - - - - - - - - - - 

which results in the combined *DSSP*-disorder string:

XXXCEEEEETTCCEEECCTTCCCCTTEEEEEEEETTEEEEEEESSCEESSTTEEECTTSCEEESSCCGGGCEEEE

EEEEETTSCEEEEEEEEEEEE

Training was performed and test accuracy was reported on all residues with known coordinates for which *DSSP* was able to assign labels, i.e. for residues with ordered ‘*-*’ flag and not with disorder ‘*X*’ flag. Each amino acid subject to training or testing includes a full amino-acid sequence around it including disordered residues.

In addition to the labels, the input to our CNN includes a 92-by-29 feature matrix where the columns represent 14 residues to the left from the central residue, the central residue which is subject to secondary structure prediction, and 14 residues to the right; the rows consist of 92 features for each position of 29 residues. Among the features are 1) one-hot encoding of 22 residue types: the 20 standard amino acids, “X” (a non-standard residue without a standard amino-acid analog), and “?” which denotes a non-existing residue outside the sequence when the central residue of an input window is too close to the beginning and end of the sequence; 2) two sets of *PSI-BLAST* sequence profiles generated against *Uniref90* sequence database at the end of the first and second rounds, each 20 long–the number of standard amino acids; 3) 30 parameters of Hidden Markov Model of the target generated by a search against the *uniprot20* sequence database computed with *hhsuite*. Thus, the number of rows in the input matrix is 22 + 2 * 20 + 30 = 92.

*PSI-BLAST* version 2.6.0 was obtained from NCBI, and used to search the *Uniref90* database of 58,284,765 sequences downloaded on Aug 2, 2017 from www.uniprot.org/downloads. *Uniref90* was converted to a *PSI-BLAST* database with the command:

*makeblastdb* -*in uniref90.fasta* -*parse_seqids* -*dbtype prot*

The *PSI-BLAST* searches were performed with the following command:

*psiblast* -*db uniref90.binary -query example.fasta -inclusion_ethresh 0.001*
-*evalue 10 -save_pssm_after_last_round -out_ascii_pssm example.mtx*
-*num_iterations 2 -num_threads 16 -save_each_pssm*

The e-value for printing out alignments is 10 but the e-value for inclusion in the profile is 0.001. *PSI-BLAST* predicts an effective search space based on the supplied e-value to speed up calculations; our approach allows us to find more hits with e-value < 0.001 but still abandons the ones above this threshold from inclusion in the profile matrix. The last flag forces *PSI-BLAST* to output each PSSM with a different file name (-1, -2, etc.). The input to *SecNet* consists of the output log-scale scores instead of rounded weighted percentages. For non-existing residues outside the complete sequence, log score values of -19 were used at each of the 20 standard amino-acid positions.

The HMM of a target vs. *uniprot20* sequence database was created with 2.0.16 *hhsuite*. *uniprot20* is located at wwwuser.gwdg.de/~compbiol/data/hhsuite/databases/hhsuite_dbs/old-releases and dated Feb, 2016. All 30 parameters of each residue in a target sequence are used from the HMM file generated with a set of two commands:

*hhblits* -*i example.fasta -d uniprot20 -oa3m example.a3m -cpu 16*
*hhmake* -*i example.a3m -o example.hhm*

The 30 parameters with ‘*’ values representing +∞ values were replaced with 99999.0. For the non-existing residues outside the *N* and *C* termini, the first 27 variables (*A*’, ‘*C*’, ‘*D*’, ‘*E*’, ‘*F*’, ‘*G*’, ‘*H*’, ‘*I*’, ‘*K*’, ‘*L*’, ‘*M*’, ‘*N*’, ‘*P*’, ‘*Q*’, ‘*R*’, ‘*S*’, ‘*T*’, ‘*V*’, ‘*W*’, ‘*Y*’, ‘*M*→*M*’, ‘*M*→*I*’, ‘*M*→*D*’, ‘*I*→*M*’, ‘*I*→*I*’, ‘*D*→*M*’, ‘*D*→*D*’) of 99999.0 value and the last 3 variables (‘*Neff*’, ‘*Neff_I*’, ‘*Neff_D*’) of 0.0 value were used for HMM parameterization. At the end all HMM parameters were divided by a normalization factor of 99,999.0.

### Training and testing a CNN for secondary structure prediction

Our neural network is a traditional CNN (Fig 2) consisting of an input layer which reads a 92-by-29 matrix centered on a residue subject to secondary structure label prediction, 4 convolutional hidden layers, densely-connected NN layer with an output dimensionality of the number of secondary structure labels, and a *softmax* activation layer returning a vector of probabilities for each label. The total number of *CNN* parameters is 2,397,960. The 4 convolutional hidden layers have the same filter length of 7 with valid border treatment and a rectified linear unit (ReLU); the number of filters at each hidden layer increases by 128 starting from 128 at the first hidden layer: 128, 256, 384 and 512. As a training regularization technique, a dropout layer with 0.30 value is implemented in front of each of the 4 hidden layers and the dense layer. This CNN was implemented and trained using 1.2.2 *Keras*, an open-source NN *Python* library with 0.8.2 *Theano* backend.

We independently trained 10 CNN models by 10-fold cross-validation on the training set. To gain additional accuracy improvement, we used a popular technique where a final predictive model is an ensemble of 10 CNNs returning a majority label vote based on 10 outputs of a predicted label. The final accuracy was reported based the independent test set that had never been used during training, validation, or making design decisions. The test set was used only once to report the final results such as the final overall accuracy, confusion matrix, and accuracy improvement for each component of the predictive model during the retrospective ablation study to update these overestimated values based on the original validation set with the unbiased ones based on the testing set. However, during algorithm development all decisions on how to train, what input features to use, what third-party software arguments to use, which sequence databases to employ and what CNN architecture to use were only based on the validation set to avoid overestimated accuracy at the very end with the testing set.

A stochastic gradient descent was used for optimization. We tried many different combinations of parameters using our optimization strategy (next section) and empirically selected the following optimization parameters leading to the best validation accuracy during many training trials: *loss function = categorical cross entropy*, *number of epochs = 650*, *batch size = 256*, *learning rate = 0*.*01*, *momentum = 0*.*9*, *Nesterov accelerated gradient = off*, *decay = learning rate / number of epochs = 0*.*01 / 650 = 1*.*54e-5* and *dropout = 0*.*3*. We computed validation accuracy at the end of each epoch and saved a model if the validation accuracy was higher than at any previous epoch disregarding a value of the training set accuracy.

A single training requires about 4 days on a 16-core CPU workstation; within the first 12–24 hours the validation accuracy was typically 0.3–0.5 points below the final best accuracy. The training was stable if different starting random seed states were used leading to a spread of final validation accuracies of only up to 0.1 points.

Training and testing were based on overall accuracy which includes all residues with assigned secondary structure different from ‘*X*’ (disordered residues with missing coordinates). The accuracy was calculated as a number of correctly predicted labels / number of all assigned labels.

### Optimization strategy

The accuracy as a function of multiple hyper-parameters is non-linear and has numerous local maxima. Training and validation of a single NN model with a set of selected hyper-parameters is a computationally expensive task of several hours on a single multiple-core CPU. The multi-dimensional space of hyper-parameters makes this problem impossible to solve exactly in a timely manner.

Our optimization strategy is a mixture of the following repeated steps in varying orders: (1) a subjective, biased search based on our domain experience and expectations, literature and forum recommendations; (2) an exhaustive search on sets of 2–4 related parameters such as (learning rate, momentum, decay) or (network depth, convolutional width) or (PSI-BLAST rounds, HMM and PSI-BLAST application parameters); (3) a random search in the multi-dimensional space with a linear analysis of prevailing trends and identifying the most critical hyper-parameters. During this multi-step procedure, we sometimes added previously unexplored hyper-parameters and sometimes eliminated some existing ones. During this process, our experience and expectations changed. In the final step, we independently and exhaustively optimized single parameter choices or pairs or triples or quadruples among all of the hyper-parameters where it was computationally possible to find a local optimum again. All of these steps were done based on the accuracy calculated from the validation data set.

Our optimization strategy is empirical and does not find a global optimum; at most it finds the best optimum among a limited number of local optima that were searched.

### Benchmarking of competitor software

We ran *DeepCNF* and *eCRRNN* with default arguments. An ensemble of 10 models was enabled for *eCRRNN*. We made sure that we were executing these third-party programs properly by benchmarking them on data sets included in their respective published studies and reproducing the same results within 0.1% accuracy for each included set. As with *SecNet*, *DeepCNF* and *eCRRNN* were benchmarked on complete original sequences and accuracy was reported on residues of known secondary structure.

## Supporting information

S1 File This file contains Fig A, Table A and Table B in S1 File.
(PDF)

S1 Dataset This tab-delimited plain text file contains *Set2018* data set which includes PDB entry codes, chain IDs, complete amino-acid sequences and ground-truth secondary structure information (*DSSP* 8 labels, *Rule #1* and *Rule #2* 3 labels, 4 labels, and 5 labels) for the training, validation, and testing (*Test2018*) sets. (TXT)
