# Supplementary material for: Multifaceted analysis of training and testing convolutional neural networks for protein secondary structure prediction
Source: PLoS One. 2020 May 6;15(5):e0232528. doi: 10.1371/journal.pone.0232528 (PMC7202669; doi:10.1371/journal.pone.0232528)
Supplement: S1 File — This file contains Fig A, Table A and Table B in S1 File. (PDF) [file pone.0232528.s001.pdf]

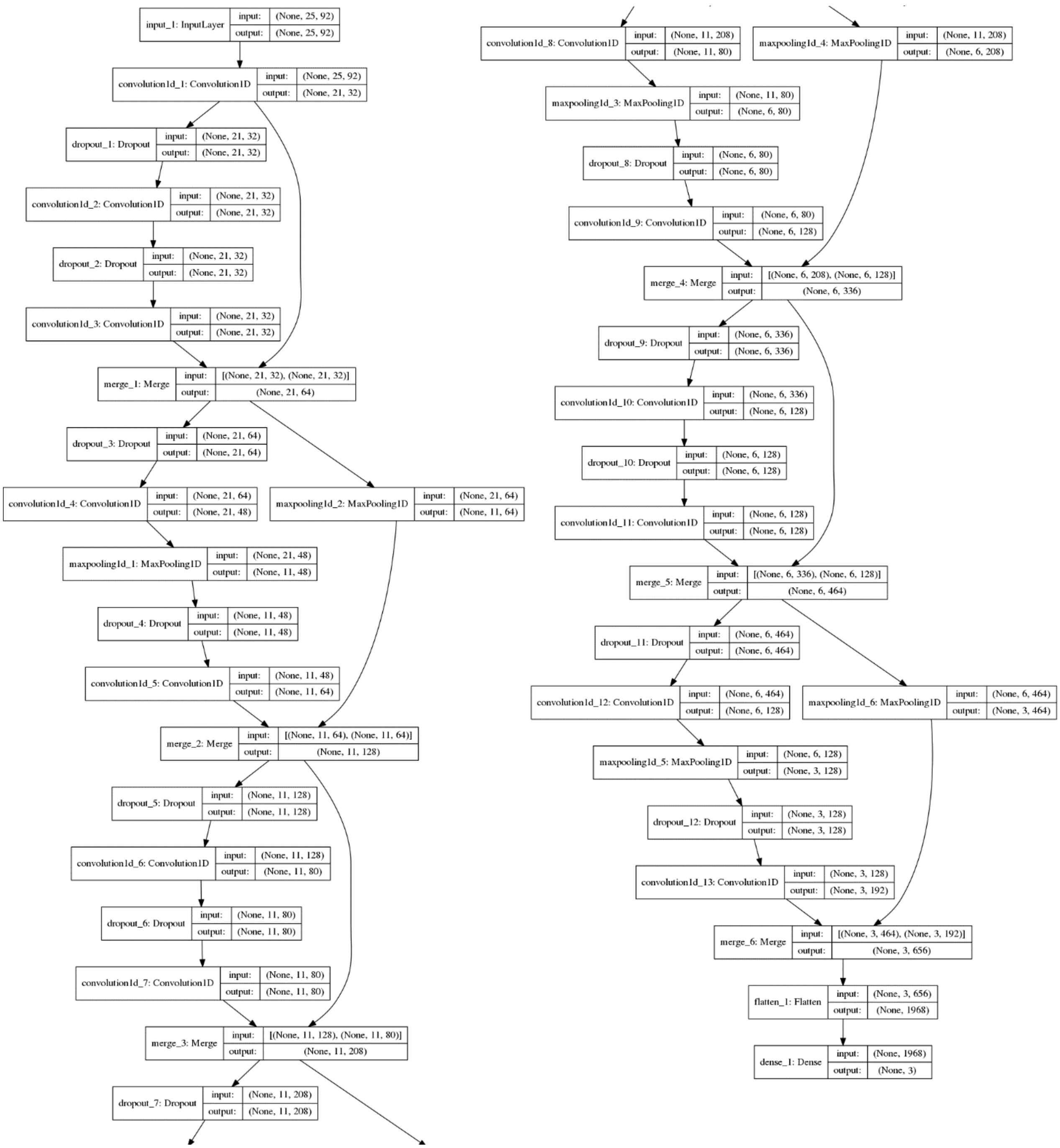

Fig A. Architecture of *SecRes*, our abandoned Residual Neural Network with input window of 21 to 51 amino acids and 20-40 convolutional layers. A sample architecture diagram of our residual neural network, *SecRes* from a family of networks with input of 21 to 51 amino acids and 20 to 40 layers. The sample has 13 hidden convolutional layers, 6 shortcut connections each bypassing 2 hidden layers and input window of 25 amino acids. Output from the blocks of 2 bypassed hidden layers is concatenated with the input to these blocks. Maxpooling of size 2 is applied for linear dimensionality reduction. Other details about the network are same as in Fig 2 about our traditional 4-layer CNN *SecNet*. We varied network complexity with a layer number, input size, and number of training parameters from 600 thousand to 20 million, observed same or worse accuracy as for *SecNet*, and as a result abandoned this more complex network.

Table A. Amino-acid frequencies in 8,712 proteins of *Set2018*.

No	Amino acid		Full sequences		Amino acids with coordinates		Freq diff
			Freq.	Count	Freq.	Count	
1	L	Leucine	9.2	204,270	9.5	196,241	-0.3
2	A	Alanine	8.1	179,432	8.1	168,536	0.0
3	G	Glycine	7.1	157,876	7.0	144,414	0.1
4	E	Glutamic acid	6.8	149,862	6.7	139,734	0.1
5	V	Valine	6.7	149,316	6.9	143,543	-0.2
6	S	Serine	6.3	140,014	6.0	125,369	0.3
7	D	Aspartic acid	5.9	131,346	5.9	123,358	0.0
8	K	Lysine	5.7	127,072	5.7	118,597	0.0
9	I	Isoleucine	5.6	123,593	5.8	119,761	-0.2
10	T	Tyrosine	5.4	120,470	5.5	113,215	-0.1
11	R	Arginine	5.1	112,709	5.1	106,255	0.0
12	P	Proline	4.6	101,358	4.5	94,171	0.1
13	N	Asparagine	4.3	96,212	4.3	89,708	0.0
14	F	Phenylalanine	4.0	89,175	4.2	86,342	-0.2
15	Q	Glutamine	3.8	84,470	3.8	78,832	0.0
16	Y	Tyrosine	3.5	78,064	3.7	75,866	-0.2
17	H	Histidine	2.9	63,656	2.4	49,127	0.5
18	M	Methionine	2.3	51,485	2.2	45,346	0.1
19	W	Tryptophan	1.4	31,152	1.5	30,453	-0.1
20	C	Cysteine	1.2	26,034	1.2	25,306	0.0
21	X	Nonstandard	0.0	141	0.0	127	0.0
**All**	**Any**	**Any**	**100.0**	**2,217,707**	**100.0**	**2,074,301**	**0.0**

Amino-acid type distributions with or without inclusion of disordered residues are almost identical. Protein sequences are represented with 21 letters of the standard one-letter notation where modified amino acids are encoded with single letters of unmodified counterparts where possible and others are denoted “X”.

Table B. Alternative 4-label alphabet: *H*, *C*, *E*, and *T* where *G* abutting *H* $\rightarrow$ *H* and rest *G* $\rightarrow$ *C*: (1) recalls and false negative rates and (2) precisions and false discover rates of *SecNet*.

**Accuracy**		**True label**			
**80.3%** Column normalized table		**H**	**C**	**E**	**T**
		100	100	100	100
**Pred. label**	**H**	**93.7**	6.1	1.2	15.6
	**C**	4.3	**78.6**	14.8	32
	**E**	0.5	10.1	**83.4**	2.1
	**T**	1.5	5.3	0.6	**50.2**
**Diagonal has TPRs** Elsewhere FNRs					

Row normalized table			**True label**			
			**H**	**C**	**E**	**T**
**Pred. label**	**H**	100	**89.3**	5.3	0.7	4.7
	**C**	100	4.5	**75.0**	9.9	10.6
	**E**	100	0.8	14.4	**83.8**	1.0
	**T**	100	6.7	21.4	1.7	**70.3**
**Diagonal has PPVs** Elsewhere FDRs						

The 4 labels are defined as: (*S*, *B*, *G* not abut *H*) $\rightarrow$ (*C*) and (*I*, *G* abutting *H*) $\rightarrow$ (*H*). This 4-label alphabet has a higher overall accuracy of 80.3% compared to 79.9% for our final 4-label alphabet (Table 11) which has *all G* $\rightarrow$ *H*. The 3-label derivative alphabet formed by *T* $\rightarrow$ *C* from this 4-label alphabet has no accuracy difference when compared to our final 3-label alphabet (Table 12). Thus this 4-label and its derivative 3-label alphabets were both rejected despite having a slightly higher accuracy of 0.4% and same accuracy respectively in the sake of the simpler rules in our final choice.
